# Engineering of CD63 Enables Selective Extracellular Vesicle Cargo Loading and Enhanced Payload Delivery

**DOI:** 10.1002/jev2.70094

**Published:** 2025-06-17

**Authors:** Wataru Obuchi, Ayrton Zargani‐Piccardi, Kevin Leandro, David Rufino‐Ramos, Emilio Di lanni, Dawn Madison Frederick, Katia Maalouf, Lisa Nieland, Tianhe Xiao, Pierre Repiton, Christine A. Vaine, Benjamin P. Kleinstiver, D. Cristopher Bragg, Hakho Lee, Miles A. Miller, Xandra O. Breakefield, Koen Breyne

**Affiliations:** ^1^ Department of Neurology Massachusetts General Hospital and Harvard Medical School Boston Massachusetts USA; ^2^ Center for Neuroscience and Cell Biology (CNC) University of Coimbra Coimbra Portugal; ^3^ Center for Genomic Medicine Massachusetts General Hospital Boston Massachusetts USA; ^4^ Department of Pathology Massachusetts General Hospital Boston Massachusetts USA; ^5^ Department of Pathology Harvard Medical School Boston Massachusetts USA; ^6^ The Collaborative Center for X‐Linked Dystonia‐Parkinsonism Massachusetts General Hospital Boston Massachusetts USA; ^7^ Department of Neurosurgery Leiden University Medical Center Leiden RC The Netherlands; ^8^ Section of Pharmaceutical Sciences, Institute of Pharmaceutical Sciences of Western Switzerland University of Geneva Geneva Switzerland; ^9^ Center for Systems Biology Massachusetts General Hospital Boston Massachusetts USA; ^10^ Department of Radiology Massachusetts General Hospital and Harvard Medical School Boston Massachusetts USA

**Keywords:** Cre recombinase, CRISPR, gene therapy, enveloped delivery vehicles, extracellular vesicles, virus‐like particles

## Abstract

Extracellular vesicles (EVs) are mediators of intercellular communication through the transfer of nucleic acids, lipids and proteins between cells. This property makes bioengineered EVs promising therapeutic vectors. However, it remains challenging to isolate EVs with a therapeutic payload due to the heterogeneous nature of cargo loading into EVs. In this study, enrichment of EVs with a desired cargo was possible through engineering of the hallmark CD63 transmembrane protein. E‐NoMi refers to engineered CD63 with mCherry on the inside of the EV membrane and a tag (3xFLAG) exposed on the outside of the EV membrane. To facilitate EV loading during biogenesis, cargo proteins, such as EGFP, Cre recombinase and the CRISPR‐Cas nuclease (SaCas9), were fused to a nanobody (Nb) protein with a high affinity for mCherry. FLAG‐tag‐based immunocapture from cell conditioned media allowed selection of cargo‐loaded E‐NoMi‐EVs, and tobacco etch virus (TEV) protease cleavage sites were used to remove the 3xFLAG‐tag from the surface of E‐NoMi‐EVs after capture. For functional payload delivery to recipient cells, the vesicular stomatitis virus G (VSV‐G) fusogenic protein was incorporated into E‐NoMi‐EVs to form fusogenic EV‐based vectors (EVVs) and proved to be 10‐fold more effective at cargo delivery than EVs generated by size‐exclusion chromatography. Functional delivery of cargo with E‐NoMi‐EVVs was validated in two mouse brain models in vivo.

## 1 Introduction

There has been a quest for advanced carriers of payloads beyond genetic cargo, particularly in the context of gene therapy and gene editing applications (Campbell et al. 2019). Despite the recent development of improved compact gene editors with robust activities for a wide range of target recognition sites with reduced off‐target effects, traditional nonintegrating vectors, such as adeno‐associated viruses (AAVs) and nanoparticles containing synthetic mRNA, still encounter challenges, including length of expression times and limited coding capacity (George et al. 2020). Although long‐term expression of gene therapeutic agents raises safety concerns, the latter constraint often requires the delivery of multiple different payloads to the same recipient cell. These obstacles limit the drive for new modes of genetic engineering in the clinic, including base editors and prime editors for precise modifications that enable insertions, deletions and substitutions in the genome (Banskota et al. 2022; Hamilton et al. 2024).

Extracellular vesicles (EVs) are naturally secreted bilayer membrane‐enclosed vesicles containing a variety of cell‐derived biomolecules. From a gene therapy point‐of‐view, EVs lack efficient payload deposition capacity (Du et al. 2022), and therefore, researchers implement viral components to modify them into effective fusogenic delivery vehicles (Hung and Leonard 2016). Vesicular stomatitis virus‐G (VSV‐G) is a viral protein that has been extensively used in gene therapy applications because it facilitates fusion at the endosomal membrane (Banskota et al. 2022; Hamilton et al. 2024; Stranford et al. 2023). VSV‐G is utilized with our EVs to aid in the delivery of cargo into the cell cytoplasm through endosomal escape (Elsharkasy et al. 2024; Hung and Leonard 2016; Liang et al. 2024; Meyer et al. 2017). We refer to our engineered EVs with VSV‐G as EV‐based vectors (EVVs). For gene editing purposes, ribonucleoproteins (RNPs) delivered through EVs could enter the cytoplasm of the recipient cell, navigate to the cell nucleus, induce edits in the genome and then degrade rapidly to minimize the risk of off‐target effects (Leandro et al. 2024) (Di Ianni et al., in press). Although EVVs have been suggested as a promising carrier for premade therapeutic payloads that can be generated by engineered cells in culture, several challenges arise from their EV scaffold: (1) different subtypes of EVs carry distinct sets of biomolecules (Kowal et al. 2016; Yáñez‐Mó et al. 2015); (2) EVs primarily transport small or fragmented cargo (O'Brien et al. 2020); (3) purifying EVs is complicated and laborious due to the presence of free proteins and other biomolecules (Ter‐Ovanesyan et al. 2021; Welsh et al. 2024); (4) competition with the “natural” cargo of EVs can hinder targeted loading efforts and (5) cargo‐loaded EVs are diluted among non‐cargo‐loaded ones.

In this study, we aimed to address some of these challenges by utilizing our previously reported NoMi construct, which was initially developed to assess diagnostic brain‐related biomarkers carried by EVs (Rufino‐Ramos et al. 2022). The term ‘NoMi’ refers to the human CD63 tetraspanin scaffold tagged with dual reporters: (1) mCherry projecting into the EV's inner surface and (2) NanoLuciferase (NanoLuc) protruding from the EV's outer surface, referred to as, NanoLuc outside—mCherry inside (No‐Mi) throughout this study. To facilitate the immunocapture of NoMi EVs from crude samples, we have incorporated a 3xFLAG‐tag on the EV's outer surface, which has proven useful for isolating and recovering distinct EV subtypes from blood (Maalouf et al. 2023; Rufino‐Ramos et al. 2022). To achieve enhanced expression of the NoMi construct, we opted to employ the human elongation‐factor 1 alpha (EF‐1α) promoter. Consequently, the construct has been designated as E‐NoMi to reflect the incorporation of the EF‐1α promoter. We have repurposed the E‐NoMi platform for gene therapy by utilizing the 3xFLAG‐tag as an enrichment handle for isolation of specifically loaded EVs and the luminal mCherry projection of E‐NoMi as a tether or cargo handle for a mCherry‐specific nanobody (Nb). This Nb has been fused with multiple‐size cargos of interest, including EGFP, Cre recombinase and *Staphylococcus aureus* Cas9 (SaCas9) (Kleinstiver et al. 2015). Alongside the versatility of recombinant cargo, the E‐NoMi selective isolation technique exhibited effective cargo transfer to recipient cells and outperformed EVVs isolated by conventional size‐exclusion chromatography (SEC). Finally, we have incorporated two tobacco etch virus (TEV) sites to remove the 3xFLAG‐tag and NanoLuc on the exterior of E‐NoMi EVVs, thereby generating an unblemished EVV as a vector.

## 2 Materials and Methods

The experimental flow was designed as follows, first we aimed to demonstrate the benefit of the immunocapture method over the conventional SEC isolation. Following confirmation, we sought to demonstrate the functionality of our payload with delivery mediated by transfection reagents. Once we confirmed the payload was functional, we incorporated a fusogen to our EVs to facilitate effective payload delivery. To build on these findings, we tested different payloads and increased the in vivo applicability by removing the immunogenic enrichment handle.

### 2.1 Gibson Assembly to Generate New Constructs

To generate EGFP fused Nb constructs, a gBlock gene fragment (Integrated DNA Technologies, Inc.) of 514 bp encoding anti‐mCherry Nb was assembled into a SalI/BsrgI restricted vector (addgene #17446) containing EGFP. Similarly, an anti‐ALFA tag Nb was assembled with a gBlock of 481 bp into the SalI/BsrgI restricted vector and used as a nonspecific Nb. The amino acid sequence of anti‐mCherry and anti‐ALFA tag nanobodies was adopted from addgene plasmids (#162276 and #171818, respectively) after codon optimization for mammalian expression with SnapGene v7.0.2 software. We used these constructs to introduce CRE to generate CRE‐Nb‐expressing plasmids. Anti‐mCherry‐Nb expressing plasmid was digested with BamHI/XcmI to introduce Cre that was amplified from an in‐house plasmid with 5′‐CACGCTGTTTTGACCTCCATAGAAGACACCGACTCTAGAGATGCCCAAGAAGAAGAGGAAGGT‐3′ and 5′‐GGCCGCCGCCAGATTCCACCAGTTGCACCTGGGCCATGGGGGGGTGTATATCGCCATCTTCCAGCAGG‐3′. Similarly, anti‐ALFA tag Nb expressing plasmid was digested with BamHI/AscI to introduce Cre that was amplified from an in‐house plasmid with 5′‐CACGCTGTTTTGACCTCCATAGAAGACACCGACTCTAGAATGCCCAAGAAGAAGAGGAAGGT‐3′ and 5′‐GCTGCACCAGGCCGCCGCCGCTCTCCTGCAGCTGCACAGGATCGCCATCTTCCAGCAGG‐3′. The SaCas9 was fused to anti‐mCherry Nb by Gibson Assembly in XhoI/NotI restricted vector provided by Dr. B. Kleinstiver (MGH) with amplicons generated with 5′‐CATTAAAAAAGGTGGATCCCCCAAGAAGAAGAGGAAAGTCTCGCTCGAGGCTAGCAcGATGGCCCAGGTGCAACTGGT‐3′ and 5′‐GAGAAGTTTGTTGCCCGACGCGTCTTGATGGCCTCCTTGCTCACG‐3′ on the above‐generated Nb containing plasmid and 5′‐TGAGCAAGGAGGCCATCAAGACGCGTCGGGCAACAAACTTCTCTCTGC‐3′ and 5′‐GCCACCACCTTCTGATAGGCAGCCTGCACCTGAGGAGTGCGGCGGCCGGCCCGCGCCACCACCTTCTGATAGGCAGC‐3′ on the original vector from Dr. B. Kleinstiver (MGH). To extract copGFP from our original E‐NoMi construct (addgene #83357) to generate the E‐NoMi‐Red plasmid, we digested with SgrDI and NheI and assembled it with a PCR product generated with 5′‐GTGAAGAGTATCAGAAGTGGCTACGAGGTGATGGAATTCTGTATGGTGAGCAAGGGCGAG‐3′ and 5′‐ACCGCATGTTAGCAGACTTCCTCTGCCCTCTCCACTGCCGTACTTGTACAGCTCGTCCATGCC‐3′ primers from the E‐NoMi construct. To generate the E‐‘No'Mi‐Red construct with TEV sites surrounding 3xFLAG‐tag and NanoLuc, we performed an XbaI digest on E‐NoMi‐Red and introduced a 762 bp gBlock (see supplemental material) through Gibson assembly. The Gibson assembly reaction was performed according to manufacture standard guidelines.

### 2.2 Production of Cargo‐Loaded EVs

HEK293T cells were plated at 50% confluency in a 15‐cm petri dish and incubated overnight in DMEM with 10% FBS and 1% penicillin–streptomycin (P/S), after which they were transfected using PEI 25K (Polysciences) and 10 µg E‐NoMi‐Red plasmid, 15 µg VSV‐G plasmid and 20 µg of cargo plasmid at 1:3 DNA:PEI ratio. After 24 h, the media was changed to 20 mL of OPTI‐Mem (1% P/S). Three days postmedia change, the conditioned cell culture media was collected and centrifuged at 400 × *g* for 10 min to remove cellular debris. For E‐NoMi stable cell line production of EVs, the same steps were followed without E‐NoMi‐Red plasmid for transfection. Due to transiently expressed E‐NoMi‐Red and anti‐mCherry Nb (EGFP) having high expression in producer cells (Figure S7a, b), as well as higher delivery efficiency that stably expressed E‐NoMi‐Red (Figure S7c), experiments proceeded using transient E‐NoMi‐Red.

### 2.3 EVs Isolation

*Immunocapture*: Twenty millilitres of culture medium was concentrated to 500 µL using Amicon Ultra 100k spin filters (6000 × *g*, 15 min) and incubated with 100 µL of anti‐FLAG M2 beads at 4°C overnight on a HulaMixer. Following incubation with magnetic immunocapture beads, E‐NoMi‐Red EVs or ‘No'Mi‐Red EVs that were pulled down onto the beads were separated from nonadhered to the beads using a magnetic rack, and the beads were washed 2x with PBS. EVs were released from beads using 3xFLAG Peptide (APExBIO) diluted in PBS for 2 h at room temperature (RT) on a HulaMixer.

*SEC*: Twenty millilitres of culture medium was concentrated to 500 µL using Amicon Ultra 100k spin filters (6000 × *g*, 15 min) and loaded onto qEV Original SEC Columns (IZON Sciences). Thirty fractions were collected using the Automatic Fraction Collector (AFC) according to the manufacturers protocol.

### 2.4 EV/EVV Incubation

For EV transfections, dose‐dependent volume of EVs (maximum of 20 µL) was combined with 1 µL Lipofectamine 2000 in 20 µL Opti‐MEM (ThermoFisher Scientific, Waltham, MA). Following a 10 min incubation of EVs and transfection reagent, the mixture was added dropwise to cells in a 96‐well plate. For EVV incubations, dose‐dependent volume of EVVs (maximum of 20 µL) was added dropwise to cells in a 96‐well plate. The cells were collected for analysis 72 h after incubation.

### 2.5 TEV Protease Recovery of ‘No'Mi‐Red EVs From Immunocapture Beads

Ten microlitres (10,000 U/mL) of TEV Protease (New England BioLabs, Ipswich, MA) was added to anti‐FLAG‐selected EVs to a final volume of 500 µL and incubated overnight at 4°C on a HulaMixer.

### 2.6 ExoDisc Purification

TEV protease‐released EVs were then filtered using the ExoDiscovery platform with an ExoDisc (LabSpinner, Ulsan, South Korea). ExoDisc purification was performed according to manufacturer's protocol. In summary, 1 mL of 0.22 µm filtered PBS was added to the chamber and run in the ExoDiscovery for 1 min and 30 s. The waste was removed, followed by the loading of up to 1 mL of our concentrated culture medium into the chamber. The ExoDisc was run in the ExoDiscovery for a minimum of 5 min or until the sample chamber had been emptied to allow for the separation of EVs from TEV protease and the FLAG‐tag handle. Once finished, the ExoDisc was washed (2x) with PBS and run until all PBS had flowed into the waste chamber to allow for thorough sample purification. The sample was eluted by adding 10 µL of PBS through the elution hole to fill the air bubble in the filter chamber. Purified EVs (100 µL) were collected through the elution hole.

### 2.7 Bioluminescence Assay

To determine the incorporation of the NanoLuc reporter derived from the E‐NoMi construct in EVs, bioluminescence was measured using Furimazine (Nano‐Glo Luciferase substrate, Promega, Madison, WI), at a dilution of 1:500 in PBS. Five microlitres of EV sample was diluted in 95 µL of PBS and added to a 96‐well white Lumitrac plate. Fifty microlitres of diluted substrate was then added to each well, and bioluminescence was measured using the Synergy H1 Hybrid Multi‐Mode Reader (Agilent, Lexington, MA).

### 2.8 EV Assessment With NTA

We performed a similar method as previously published (Maalouf et al. 2023). In short, EV samples were diluted with 1x PBS to a final volume of 500 µL. Settings were adjusted according to the manufacturer's software manual (NanoSight LM10 and NTA 3.2): screen gain and camera level were increased until all particles were distinctly visible. For each measurement, five 1‐min videos were captured, after which all the videos were analysed by the in‐built NanoSight Software NTA 3.2 with a screen gain of 6 and detection threshold of 2.

### 2.9 ExoView Assessment of EVs

Single‐EV analysis using ExoView was performed according to the guidelines provided by Unchained Labs. In short, an ExoView Tetraspanin Chip with anti‐CD63, anti‐CD81 and anti‐CD9 antibodies on the chip surface was prescanned for a baseline measurement. EVs were then incubated overnight on the chip. After washing the chip (3x) with the company‐provided wash buffers, cargo imaging of endogenous mCherry and EGFP was performed with the ExoView R100 reader. As we were detecting endogenous fluorescent cargo of our EVs, no detection antibodies were used.

### 2.10 RT‐qPCR Gene Expression Analysis

cDNAs for gene expression analysis with RT‐qPCR were prepared using the SuperScript VILO cDNA Synthesis Kit (Invitrogen). qPCR mix was prepared following the manufacturing protocol of Power SYBR Green PCR Master Mix (Applied Biosystems). qPCR was performed using the QuantStudio 3 PCR system (Applied Biosystems). The cycling conditions used were 50°C for 2 min, 95°C for 10 min and 40 cycles of 95°C for 15 s and 60°C for 1 min following dissociation analysis. All qPCR reactions were done in triplicate and normalized to the housekeeping gene β‐actin or GAPDH mRNA levels. A list of primers used can be found in Table S1.

### 2.11 Flow Cytometry

Reporter cells incubated with E‐No‐Mi EVs/EVVs were trypsinized and centrifuged at 300 × *g* for 5 min. Samples were resuspended in 1% FBS, 5 mM EDTA in dPBS, and passed through a round‐bottom polystyrene tube with a 35 mm cell Strainer (Fisher Scientific). Cells were run through a BD Accuri C6 flow cytometer with an appropriate laser for each fluorophore (iRFP) according to the manufacturer's instructions. Percentages of iRFP+ cell populations were analysed with FlowJo software version 10.10.0 (https://www.flowjo.com).

### 2.12 Western Blot

2.0 × 10^9^ E‐NoMi‐Red EVs or 2 µg of cell lysate, diluted to 30 µL, was mixed with 10 µL of Laemmli SDS‐Sample buffer (Boston BioProducts, Milford, MA) and loaded on NuPage 4%–12% Bis‐Tris polyacrylamide gels (ThermoFisher Scientific) in NuPage MES SDS Running Buffer (ThermoFisher Scientific). Transfer onto a nitrocellulose membrane was facilitated using the iBlot2 (ThermoFisher Scientific), and the membrane was blocked using 2.5% nonfat dry milk (Lab Scientific, Danvers, MA) in Tris‐buffered Saline (pH 7.4). Tris‐buffered saline with 0.05% Tween 20 (TBS‐T) was used for primary antibody probing. The antibody probing solution was prepared with a dilution of 1:1000 in TBS‐T supplemented with 10% nonfat milk solution and incubated overnight at 4°C on a shaker. The membrane was washed three times, for 5 min each, with TBS‐T, then incubated for 1 h with secondary antibody (1:3000) and rewashed. Membranes were developed with Femto staining (ThermoFisher Scientific) and imaged using an Azure Biosystems C300 gel imager. A list of primary antibodies can be found in Table S2.

### 2.13 SaCas9 Experiments

Cells were transfected with pDNA of the *EMX1* sgRNA in a 24‐well plate. 300 ng of pDNA was transfected to HEK293T cells at 75% confluency using 2 µL of Lipofectamine 2000 (ThermoFisher Scientific). After 24 h, cells were counted and seeded in a 96‐well plate for EV incubation. For SaCas9 pDNA control, cells were reseeded in a 24‐well plate and transfected with 300 ng of SaCas9‐Nb pDNA. Coloading of sgRNA and SaCas9 in our EVs was accomplished with an additional 10 µg of sgRNA plasmid included during the transfection of our EV‐producer cells. *EMX1* sgRNA sequence can be found in Table S3.

### 2.14 Amplicon Sequencing and CRISPR Analysis

CRISPR analysis was performed in accordance with a previously published two‐step PCR‐based Illumina library construction protocol (Ferreira Da Silva et al. 2024; Walton et al. 2020).

Briefly, the genomic locus of interest was amplified from genomic DNA (gDNA) using Q5 High‐fidelity DNA Polymerase (New England Biolabs). A second PCR was done to add barcodes and Illumina adapter sequences. Final libraries were sequenced with MiSeq sequencer. Final amplicon sequencing data was analysed using CRISPResso2 to determine on‐target genome editing (Clement et al. 2019).

### 2.15 Stereotactic Intracranial Injection Into the Mouse Brain

Mice (Protocol #: 2009N000054) were anesthetized using 2.5% isoflurane in 100% oxygen via a nose cone. With reference to the bregma, mice were stereotactically injected into the left striatum with the following coordinates: anteroposterior: +0.52 m, medial‐lateral: +2.00 mm, dorsal‐ventral: −2.5 mm. A total of 200,000 hNPCs or 25,000 CT‐2As were resuspended in a volume of EVVs equivalent to 1 × 10^5^ EVVs/cell and diluted to a final volume of 2 µL with PBS. The cells were implanted at an infusion rate of 0.25 µL/min using a 10‐mL 26s Gauge Hamilton syringe (Reno, NV). Five minutes after the infusion was completed, the needle was retracted 0.3 mm and allowed to remain in place for an additional 3 min before complete removal from mouse brains.

### 2.16 Mouse Brain Tissue Preparation for Immunofluorescence and RNA Extraction

Fouurteen/seventeen days postinjection of transduced NPCs and CT2As, the mice were euthanized under deep anaesthesia induced by a combination of xylazine and ketamine (10 and 100 mg/kg, respectively) as previously reported (Xu et al. 2007). For immunofluorescence, mice underwent cardiac perfusion with 1x PBS, followed by 4% PFA, and brains were harvested and cryopreserved at −80°C using optimal cutting temperature (OCT) compound (Neg‐50 #6502, Expredia, Kalamazoo, MI) for coronal sectioning onto glass slides. Coronal sections of mouse brains were obtained using a freezing cryostat (Leica Microsystems, CM3050S, Deer Park, IL) and mounted on slides.

In the case of RNA extraction, mouse brains were promptly flash‐frozen and preserved on dry ice. Brain sections around the injection site were coronally sectioned with sections of 100 µm thickness. Sections were homogenized in 200 µL of Nano‐Glo Luciferase substrate (Promega). Thirty microlitres was taken for bioluminescent imaging to track hNPCs. The rest of the sample was utilized for RNA extraction using RNeasy Micro Kit (Qiagen, Valencia, CA). The following qPCR was conducted with human‐specific primers to ensure that the amplification only occurred on the human cells.

### 2.17 Tissue Digestion

Tumour‐bearing (CT‐2A‐BFP/iRFP) mice were transcardially perfused with 50 mL Dulbecco's phosphate‐buffered saline (DPBS) after intraperitoneal injection of a mixture 100 µL ketamine (17.5 mg/mL) (Patterson Veterinary) and xylazine (2.5 mg/mL) (Patterson Veterinary). To process the brain into a single‐cell suspension, Tumor Tissue Dissociation Kit (Miltenyi Biotec, Charlestown, MA) was used. The ipsilateral hemisphere of the brains with 2.35 mL RPMI 1640 containing Enzymes D (100 µL), R (30 µL) and A (3.5 µL) was placed into a GentleMacs C‐tube (Miltenyi Biotec) and incubated for 40 min at 37°C. According to the manufacturer's protocol, the brains were dissociated using the gentle MACS Dissociator (Miltenyi Biotec). Samples were run through a 70 µm filter to obtain a single‐cell suspension. Magnetic separation with antimyelin beads (Miltenyi Biotec) was used for myelin removal. The final cell suspension was resuspended in 1X DPBS without calcium (Ca^2+^) or magnesium (Mg^2+^) (Corning, Corning, NY), supplemented with 2 mM EDTA (Thermo Fisher Scientific) and 0.5% BSA (MilliporeSigma, Burlington, MA). Single cells were further processed by qPCR after RNA extraction using the RNeasy Micro Kit (Qiagen).

### 2.18 Immunofluorescence

Brain sections underwent postfixation with 4% PFA for 20 min, followed by three washes with 1x PBS and a 30‐min incubation in blocking solution (PBS with 0.1% Triton X‐100 [USB #22686, Cleveland, OH, USA]) containing 10% normal goat serum (MilliporeSigma). Subsequently, the sections were incubated overnight at 4°C in blocking solution with primary antibodies: FITC anti‐GFP (Abcam, Cambridge, MA, ab6662, 1:200), rabbit anti‐RFP (Abcam, ab62341, 1:400) and Rat anti‐KI67 (Invitrogen, SolA15, 1:400). After three washes with PBS, the sections were incubated for 1 h at RT with the appropriate secondary antibodies: goat antirabbit IgG (Invitrogen, A‐21428, 1:1000), goat antirat IgG (Invitrogen, A‐21247, 1:1000) and goat antimouse (Invitrogen, A28175, 1:1000) diluted in blocking solution. Following another wash with 1x PBS, the sections were mounted on glass slides using Vectashield Antifade Mounting Medium without DAPI (Vector Labs, H‐1000, Burlington, CA) or ProLong Diamond Antifade Mountant (Invitrogen, P36965). Immunofluorescence was observed and captured using a NIKON CSU‐W1 spinning disc confocal microscope.

### 2.19 Statistical Analysis

Statistical analyses were performed using GraphPad Prism (version 10.2.1, GraphPad Software). Results are expressed as mean ± standard deviation (SD). *p* values were determined as statistically significant according to the following criteria: *p* > 0.05 = not significant (ns); *p ≤ 0.05, **p < 0.01 ***p < 0.001 and ****p < 0.0001.

## 3 Results

### 3.1 Engineering of Anti‐mCherry Nb Fused Payloads for Efficient Enrichment Into EVs

**FIGURE 1:**
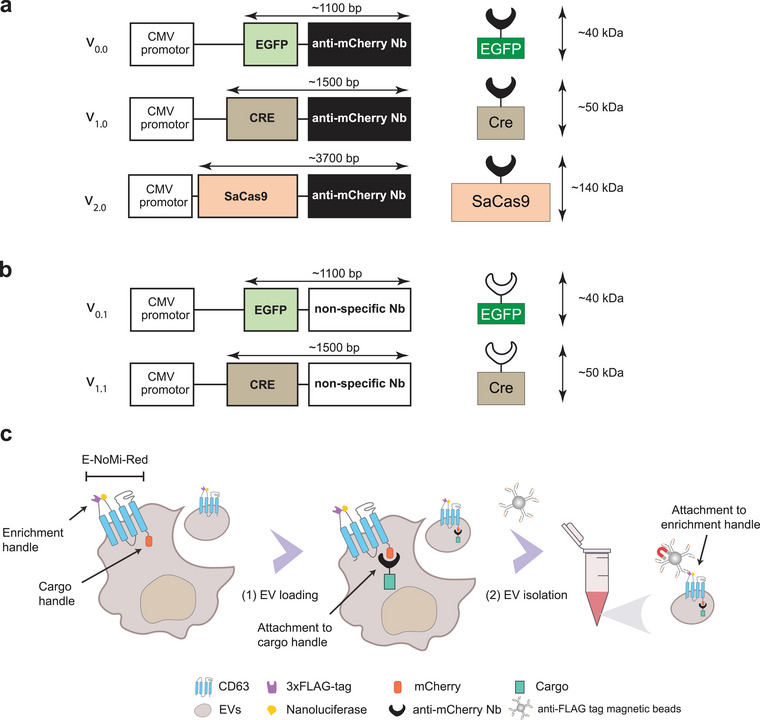
Engineering of anti‐mCherry nanobody fused payloads for efficient enrichment into EVs. (A) Constructs encoding anti‐mCherry Nb‐fused payloads. Multiple protein cargos, including EGFP (v_0.0_), Cre recombinase (v_1.0_) and SaCas9 (v_2.0_), were fused to an anti‐mCherry Nb. (B) Control designs encoding nonspecific Nb‐fused payloads. Transgene variants were generated with EGFP (v_0.1_) and Cre (v_1.1_) recombinase fused to an Nb that does not target mCherry. (C) Strategy using the CD63‐based E‐NoMi‐Red platform to enrich payload‐loaded EVs. Cells expressing E‐NoMi‐Red transgenes secrete cargo‐loaded E‐NoMi‐Red EVs. Anti‐mCherry Nb‐fused cargo expressed in E‐NoMi‐Red cells has affinity for the mCherry (cargo handle) of E‐NoMi‐Red facing the cytosol of the E‐NoMi‐Red expressing cells. Subsequently, cargo‐loaded E‐NoMi‐Red EVs can be isolated using a one‐step procedure with bead‐based immunocapture targeting the 3xFLAG‐tag (enrichment handle) of E‐NoMi‐Red, which effectively eliminates contaminants like free proteins and EVs without cargo loading. Of note, the term ‘E‐NoMi‐Red’ refers to the genetic construct, where ‘E’ denotes the EF‐1α promoter and the term ‘NoMi‐Red’ for the mCherry in the EVs and the NanoLuc on the outside. EF‐1α, elongation‐factor 1 alpha; EV, extracellular vesicle; Nb, nanobody.

To explore whether the E‐NoMi construct (pCDH‐EF1‐E‐NoMi‐P2A‐CopGFP‐T2A‐PuroR, addgene #207805) can be used as a tool to enrich payload‐carrying EVs, plasmids were designed with transgenes encoding different protein cargos fused to an anti‐mCherry Nb; EGFP (v_0.0_), Cre recombinase (v_1.0_) and SaCas9 (v_2.0_) (Figure 1a). Transgene variants EGFP (v_0.1_) and Cre (v_1.1_) fused to an anti‐ALFA tag Nb that does not target mCherry were generated as controls and referred to hereafter as nonspecific Nbs (Figure 1b). We also removed the P2A‐copGFP element from the E‐NoMi construct, ensuring that copGFP (Copepod green fluorescent protein) fluorescence would not interfere with the assessment of EGFP loading in the Nb‐fused cargo (see section 3.2). The term ‘E‐NoMi‐Red’ refers to the genetic construct without the copGFP transgene, where ‘E’ denotes the EF‐1α promoter, and the term ‘NoMi‐Red’ highlights the presence of mCherry at the inner‐membrane side of the EVs to serve as a tether for mCherry Nb binding. With the CD63‐based E‐NoMi‐Red construct, a strategy for isolating E‐NoMi‐Red EVs and EVVs was developed (Figure 1c). Anti‐mCherry Nb‐fused cargo and the E‐NoMi‐Red loading platform were introduced into EVs/EVVs through transgene expression plasmids or lentiviral vectors in EV/EVV‐producer cells. Subsequently, we isolated EVs/EVVs from the cell media using a two‐step procedure, first employing fast centrifugal filters to generate a high‐concentration EV suspension, then bead‐based 3xFLAG‐tag immunocapture. This effectively eliminates contaminants, such as free proteins and EVs/EVVs, without the designated cargo. A commercial high‐affinity 3xFLAG‐tag peptide (DYKDDDDK) enabled the recovery of the EVs/EVVs from the FLAG‐tag immunocapture beads.

### 3.2 E‐NoMi‐Red Promotes the Enrichment of EVs Loaded With Anti‐mCherry Nb Fused Payloads

**FIGURE 2:**
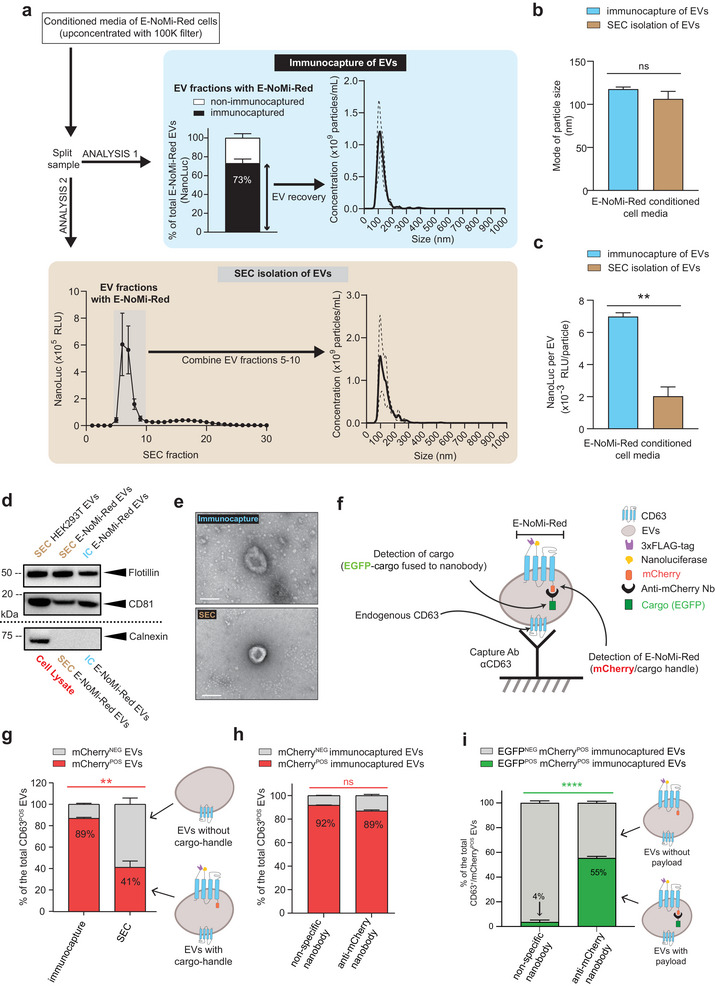
E‐NoMi‐Red promotes the enrichment of EVs loaded with anti‐mCherry Nb fused payloads. (A) Isolation of E‐NoMi‐Red EVs through anti‐FLAG‐tag immunocapture (Analysis 1) and SEC (Analysis 2). NanoLuc bioluminescence measurement on anti‐FLAG‐tag affinity beads (immunocaptured) and in suspension (nonimmunocaptured) was measured and illustrated as a percentage of total bioluminescence. SEC was followed by NanoLuc measurements in each fraction. The NanoLuc signal originating from E‐NoMi‐Red is mainly observed in SEC Fractions 5–10, indicating the presence of EVs within these fractions. EVs isolated through the different methods were also analysed using NTA. Bioluminescence data and NTA data represent measurements’ mean with SD (error bars) generated from three independent experiments. (B) Particle size of EVs. Mode of E‐NoMi‐Red EVs isolated by SEC and immunocapture. NTA measurement represents the mean with SD (error bars) and is generated from three independent experiments. (C) NanoLuc per EV (RLU/particle). The NanoLuc signal represents the number of E‐Nomi‐Red^POS^ EVs in a sample. NanoLuc levels were compared between E‐NoMi‐Red conditioned media isolated with SEC or immunocapture. Data represent mean with SD (error bars) and are generated from three independent experiments. (D) Western blot analysis of E‐NoMi‐Red EV solutions isolated by immunocapture or SEC and wild‐type HEK293T EVs showing flotillin‐1 and CD81. Another blot with E‐NoMi‐Red EVs and the cell lysate shows the cellular marker calnexin. (E) Representative TEM images of E‐NoMi‐Red EVs isolated by either immunocapture or SEC, showing round cup‐shape morphology. The scale bar represents 100 nm. Direct magnification is 30,000x. (F) Cartoon illustrating how to assess loading by Nb tethered cargo in E‐NoMi‐Red^POS^ EVs. ExoView chips have capture antibodies to bind CD63^POS^ EVs and analyse their fluorescence. Red fluorescence (mCherry^POS^) indicates E‐Nomi‐Red^POS^ EVs, and green fluorescence (EGFP^POS^) represents loading with a specific Nb (construct v_0.0_) or a nonspecific Nb (construct v_0.1_). (G) The number of E‐NoMi‐Red^POS^ EVs in an EV solution. EV suspensions generated with SEC or immunocapture were assessed for red fluorescence with an ExoView chip. Bars represent mCherry^POS^ EVs captured by immobilized anti‐CD63. Data derived from single‐EV counting of three independent spots on ExoView chip are illustrated as a percentage of all CD63^POS^ EVs discriminated based on anti‐CD63 detection antibody. (H) E‐NoMi‐Red^POS^ EV enrichment is not compromised by endogenous Nb‐fused cargo loading. EVs were isolated with immunocapture from conditioned media derived from E‐NoMi‐Red expressing cells expressing Nb‐fused EGFP (construct v_0.0_ and construct v_0.1_). The number of EVs with red fluorescence (mCherry^POS^), based on ExoView chip single CD63 ^POS^EV counting (see D) of three independent spots, was compared between cells expressing construct v_0.0_ (left bar) and construct v_0.1_ (right bar). (I) Cargo loading is improved with anti‐mCherry Nb‐fused cargo in E‐NoMi‐Red^POS^ enriched EVs. EVs assessed in (F) were analysed for green fluorescence (EGFP^POS^) to compare loading efficiencies between cargo fused to a specific Nb (construct v_0.0_) or a nonspecific Nb (construct v_0.1_). The bars represent the percentage of CD63^POS^mCherry^POS^EGFP^POS^ EVs derived from three independent ExoView chips. (Statistics) The data presented in this figure are depicted as the mean ± SD (error bars) obtained from normalized data across three independent replicates. Statistical analysis was performed using an unpaired *t* test and GraphPad Prism 10.2.1 software. **p < 0.01 and ***p < 0.001. EV, extracellular vesicle; Nb, nanobody; NTA, nanoparticle tracking analysis; SD, standard deviation; SEC, size‐exclusion chromatography.

To test our hypothesis of enriching Nb‐fused cargo‐loaded EVs/EVVs from complex biofluids, the media of E‐NoMi‐Red expressing cells was concentrated with 100k centrifugal filters. To control variability, cell media from E‐NoMi‐Red and construct v_1.0_ Cre expressing cells were harvested and split in two equal parts to better compare Cre‐loaded EVs isolated by SEC or immunocapture and avoid variability that may be introduced across transfection batches. Isolated EVs through bead‐based anti‐FLAG‐tag immunocapture or SEC (Analysis 1 and Analysis 2, respectively, in Figure 2a). NanoLuc displayed on the surface of E‐NoMi‐Red EVs of the 3xFLAG‐tag was used as a readout for measuring E‐NoMi‐Red‐expressing EVs. We first isolated ∼73% of E‐NoMi‐Red EVs upon 3xFLAG‐tag immunocapture methodology (Analysis 1). This percentage was generated by comparing the NanoLuc signal retained on anti‐FLAG‐tag affinity beads compared to the nonretained NanoLuc signal in suspension. Second, our SEC processed sample (Analysis 2) detected the NanoLuc signal in SEC Fractions 5–10, corresponding to SEC EVs. In contrast, the NanoLuc signal was less pronounced in the subsequent SEC Fractions 11–17, which primarily contain smaller molecules from the cell media, including proteins. The highest NanoLuc signal was detected in SEC Fractions 6–7. The mean NanoLuc signal at the EV peak is 14.74‐times higher (*p* < 0.01) than the peak signal in the non‐EV SEC fractions (SEC Fractions 16–17), suggesting that the E‐NoMi‐Red recombinant proteins are mainly incorporated in intact EVs from the cell culture secretome. We subsequently performed nanoparticle tracking analysis (NTA) to evaluate size and particle concentration on EVs collected from immunocapture and SEC isolation (Figure 2b). Immunocaptured EVs were dissociated from the beads using 3xFLAG‐elution peptide, while SEC EVs were obtained through pooling SEC Fractions 5–10. Both methods generated EVs with a similar mode of 106 ± 9 nm (mean ± SD) based on NTA readings. Interestingly, EVs isolated through the immunocapture method contained 3.4‐times more NanoLuc signal than SEC EVs, suggesting the immunocapture method enriched for E‐NoMi‐Red expressing EVs (Figure 2c). As expected, immunocapture of E‐NoMi‐Red EVs demonstrated similar protein concentrations of the EV markers CD81 and flotillin‐1 compared to SEC isolated E‐NoMi‐Red EVs and EVs isolated from wild‐type HEK293T cells (Figure 2d and Figure S1a). Additionally, both immunocaptured and SEC isolated E‐NoMi‐Red EVs lacked the cellular marker calnexin. Regarding EV morphology, transmission electron microscopy (TEM) analysis of immunocapture and SEC isolations show cup‐shaped structures with size in the 100 nm range, consistent with typical EV morphology (Figure 2e). To further assess enrichment, we used the ExoView platform and quantified E‐NoMi‐Red EVs with mCherry fluorescence isolated through SEC or FLAG‐tag bead‐based immunocapture (Figure 2f). EVs bound to the ExoView chip were visualized using anti‐CD63 detection antibodies. We observed comparable quantities of CD63^POS^ EVs in both SEC and immunocapture conditions, ensuring sample comparability. However, the SEC‐isolated sample contained significantly less CD63^POS^mCherry^POS^ EVs than EVs obtained through the immunocapture method (41% compared to 89%, respectively, Figure 2g). The immunocapture sample not only had substantially more CD63^POS^mCherry^POS^ EVs but also contained a three‐times increase in relative fluorescence intensity (RFI) of mCherry per EV, indicating a higher number of relative mCherry units per EV (Figure S1b).

Next, we investigated whether immunocapture could effectively enrich EVs carrying anti‐mCherry Nb‐fused cargo. E‐NoMi‐Red EVs were isolated via immunocapture from E‐NoMi‐Red cells expressing either EGFP fused to an anti‐mCherry Nb (v_0.0_) or a nonspecific Nb (v_0.1_). Both samples contained 80%–90% CD63^POS^mCherry^POS^ EVs, indicative of a similar number of E‐NoMi‐Red EVs captured by the ExoView chips and suggesting that the expression of Nb‐tethered cargo did not affect E‐NoMi‐Red EV secretion and isolation (Figure 2h and Figure S1c). To gauge the level of Nb‐mediated cargo loading into E‐NoMi‐Red EVs, we quantified the number of CD63^POS^mCherry^POS^EGFP^POS^ EVs, whereby GFP fluorescence in EVs indicates successful EGFP loading (Figure 2i). Based on EGFP fluorescence of E‐NoMi‐Red EVs, the percentage of vesicles loaded using the anti‐mCherry Nb (v_0.0_) was over 13‐times higher than vesicles loaded with the nonspecific Nb (v_0.1_), at 55.3% ± 1.5% and 3.6% ± 1.7%, respectively. Next to the number of E‐NoMi‐Red EVs loaded with EGFP through Nb affinity, we also detected higher EGFP RFI per EV (Figure S1d). This estimate of the relative EGFP units per EV was three‐times higher with the anti‐mCherry Nb (v_0.0_) compared to the nonspecific Nb (v_0.1_). It is important to note that although we enriched our EGFP‐loaded EVs with our immunocapture technique, our preparation still contained 45% of EVs that lacked EGFP loading.

In conclusion, our data illustrates that an enrichment handle (i.e., 3xFLAG‐tag) displayed on the surface of the EV can aid in selecting EVs with Nb‐fused cargo from a complex biofluid, such as cell media.

### 3.3 Quantitative Assessment of Functional Cargo Loading Into E‐NoMi‐Red EVs

**FIGURE 3:**
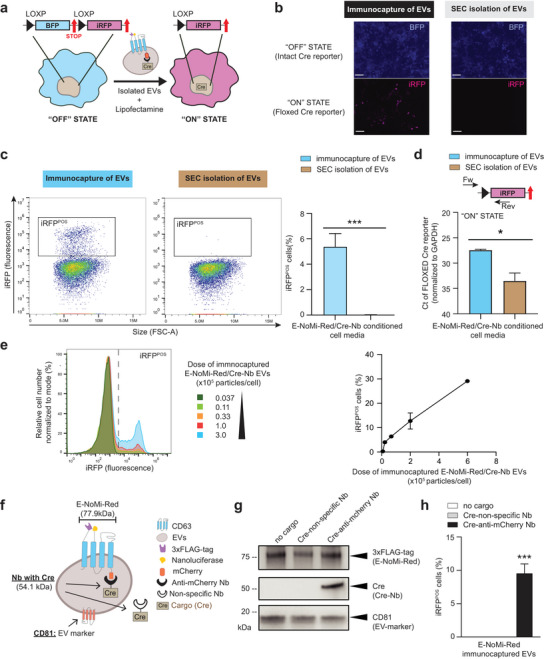
Quantitative assessment of functional Nb‐fused cargo loaded into E‐NoMi‐Red EVs. (A) Illustration describing Cre‐reporter cells that change colour from blue (OFF STATE) to far‐red (ON STATE) fluorescence upon successful Cre delivery. This model tests the activity of Cre recombinase loaded into EVs by transfecting Cre‐loaded EVs with Lipofectamine into Cre‐reporter cells. (B) Nb‐fused Cre recombinase loaded into immunocaptured E‐NoMi‐Red EVs is functional. Cre‐reporter cells were transfected with E‐NoMi‐Red EVs loaded with Cre fused to the specific Nb (construct v_1.0_). Cre‐loaded E‐NoMi‐Red EVs were isolated from conditioned media using FLAG‐tag immunoaffinity capture or SEC, and Cre‐reporter cells were evaluated through fluorescent microscopy. 3.0 × 10^4^ particles/cell were transfected into Cre‐reporter cells. The scale bar represents 100 µm. (C) E‐NoMi‐Red‐based immunocapture is more effective in enriching Cre recombinase‐loaded EVs than SEC. Flow cytometry of Cre‐reporter cells confirmed fluorescent imaging in (B). Flow plots illustrate far‐red fluorescent (iRFP^POS^) cells representing the ON STATE of the Cre‐reporter in cells exposed to either immunocaptured EVs (left dot plot) or SEC‐isolated EVs (right dot plot). The bar graphs on the right represent the mean with SD (error bars) from three independent flow cytometry experiments. (D) Validation of observations in (B) and (C) on the genomic level. RT‐qPCR of cDNA extracted from Cre‐reporter cells transfected with Cre‐loaded E‐NoMi‐Red EVs showed increased ON‐state in immunocaptured (blue bar) compared to the SEC (brown bar) condition. PCR strategy is illustrated above the bar graphs, and the bar graphs represent the mean with SD (error bars) from three independent flow cytometry experiments. (E) Dose‐dependent response of cargo‐loaded immunocaptured E‐NoMi‐Red EVs. The Cre‐reporter cells were treated with increasing doses of Cre‐loaded E‐NoMi‐Red EVs (3.7 × 10^3^, 1.1 × 10^4^, 3.3 × 10^4^, 1.0 × 10^5^ and 3.0 × 10^5^ particles/cell). Flow cytometry histograms (left) quantified far‐red fluorescent (iRFP^POS^) cells representing the ON STATE of the Cre‐reporter. The XY plot (right) represents the mean with SD (error bars) from three independent flow cytometry experiments. (F) Graphical representation illustrating all the components in our Cre‐loaded E‐NoMi‐Red EVs and the predicted molecular weights in kDa. (G) Loading cargo into E‐NoMi‐Red EVs is more efficient with specific than nonspecific Nb‐fused cargo. EVs isolated with immunocapture from media derived from E‐NoMi‐Red‐expressing cells, both with and without cargo, were applied to western blotting. E‐NoMi‐Red band (77.9 kDa) was detected in all three immunocaptured EV samples to a similar extent by immunoblot against FLAG‐tag. Immunoblot against Cre showed Cre protein only in EVs when it was fused to a specific Nb (construct v_1.0_) and not with the nonspecific Nb (construct v_1.1_). (H) Specific Nb‐fused cargo contains more active Cre recombinase than nonspecific Nb‐fused cargo in E‐NoMi‐Red EVs. Flow cytometry data that quantifies the number of far‐red fluorescent (iRFP^POS^) cells representing the ON STATE of the Cre‐reporter after adding immunocaptured E‐NoMi‐EVs. E‐NoMi‐EVs are not loaded (white), loaded through nonspecific Nb (construct v_1.1_), or specific Nb (construct v_1.0_). (Statistics) The data presented in this figure are depicted as the mean ± SD (error bars) obtained from normalized data across three independent replicates. Statistical analysis was performed using an unpaired *t* test (in C and D) or one‐way ANOVA (in H) and GraphPad Prism 10.2.1 software. *p < 0.05, **p < 0.01 and ****p < 0.0001. ANOVA, analysis of variance; EV, extracellular vesicle; Nb, nanobody; SD, standard deviation; SEC, size‐exclusion chromatography.

To assess if recombinant proteins loaded in E‐NoMi‐Red EVs can be functionally delivered, we generated a construct encoding Cre recombinase tethered to a specific, anti‐mCherry Nb (v_1.0_) and isolated EVs from cells that expressed this construct to test them with reporter cells that change colour from blue fluorescence (BFP) to near‐infrared, far‐red fluorescence (iRFP) upon successful Cre recombination (Nieland et al. 2024) (Figure 3a). Once again, to control for transfection variability, cell media from E‐NoMi‐Red and anti‐mCherry Nb‐fused Cre (construct v_1.0_) expressing cells were collected and split in two equal parts to better compare Cre‐loaded EVs isolated by SEC or immunocapture, and to avoid variability that may be introduced across transfection batches. EVs from both isolation methods were transfected into the Cre‐reporter cells at a dosage of 3.0 × 10^4^ particles/cell (based on NTA readings) with Lipofectamine 2000. Interestingly, only immunocaptured EVs generated a detectable iRFP signal in recipient cells as determined visually by fluorescent microscopy (Figure 3b) and flow cytometry (Figure 3c and Figure S2a). Genetic changes due to the Cre activity delivered by EVs were confirmed through highly sensitive RT‐qPCR analysis with primers distinguishing the recombined (ON) and nonrecombined (OFF) states of the reporter cell line (Figure 3d and Figure S2b). To determine the minimum number of Cre‐loaded E‐NoMi‐Red EVs required for a functional effect on recipient cells, we transfected immunocaptured E‐NoMi‐Red EVs with increasing doses of Cre‐loaded E‐NoMi‐Red EVs (ranging from 3.7 × 10^3^ to 3.0 × 10^5^ particles/cell as estimated by NTA). Fluorescent imaging demonstrated iRFP signal in all conditions that received more than 3.7 × 10^3^ EVs/cell (Figure S2c), which was validated by flow cytometry (Figure 3e) and RT‐qPCR (Figure S2d). Moreover, we evaluated whether Cre tethering to anti‐mCherry Nb would be advantageous for the loading into E‐NoMi‐Red EVs. We compared construct v_1.0_ (anti‐mCherry Nb) with construct v_1.1_, representing Cre tethered to a nonspecific Nb control. (Figure 3f). We observed that Cre loading in E‐NoMi‐Red EVs was only detectable with western blot analysis when using the anti‐mCherry Nb, and that the low levels of Cre passively loaded with the nonspecific Nb were not likely to be detectable with western blot (Figure 3g and Figure S2e). This result suggests that merely overexpressing Cre in cells is insufficient for producing Cre‐loaded EVs. Additionally, it suggests that we are transferring recombinant Cre protein rather than Cre‐encoded RNA or DNA, as nonspecific Nb‐expressing cells should load these latter biomolecules into EVs to a similar extent as our anti‐mCherry Nb. We confirmed these observations by transfecting immunocaptured E‐NoMi‐Red‐EVs derived from cells coexpressing Cre tethered to nonspecific Nb and anti‐mCherry Nb, as well as transfecting unloaded E‐NoMi‐Red EVs. As expected, we detected significantly more iRFP‐positive (iRFP^POS^) cells and recombined events in the anti‐mCherry Nb compared to the other conditions (Figure 3h and Figure S2f). In conclusion, our data demonstrates that enriching functional cargo‐loaded EVs is feasible with our E‐NoMi‐Red construct combined with constructs encoding functional payloads fused to an anti‐mCherry Nb.

### 3.4 Fusogenic Proteins Are Essential for Enabling Efficient Cargo Delivery of E‐NoMi‐Red‐Containing EVs Loaded With Cre and SaCas9

**FIGURE 4:**
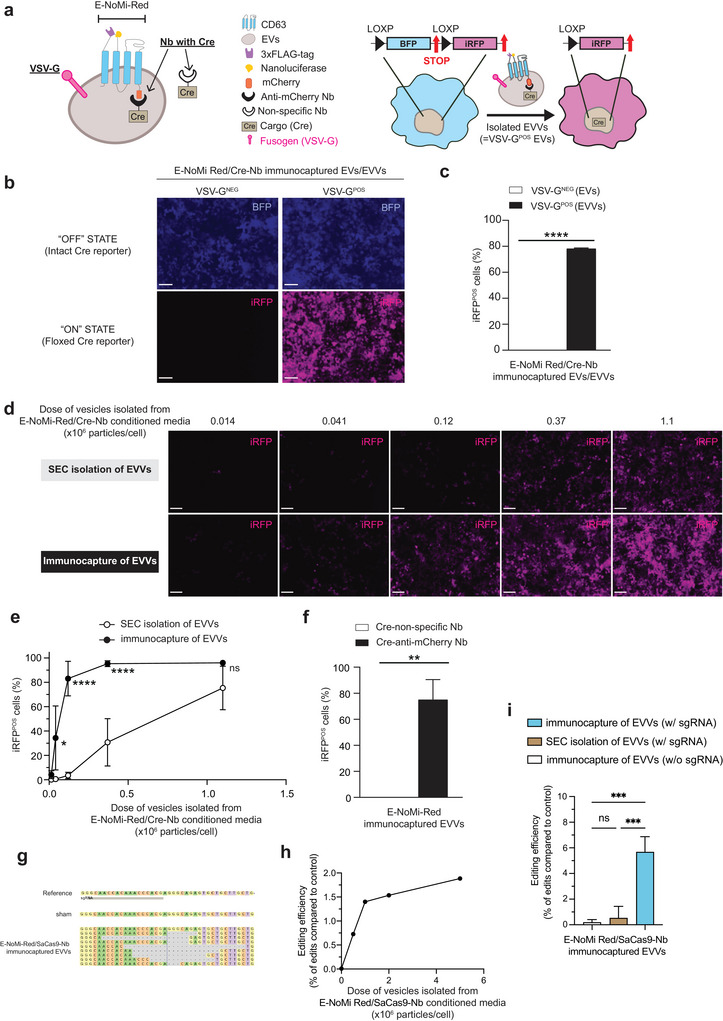
E‐NoMi‐Red EVVs are effective gene therapy carriers. (A) Cartoon illustrating the addition of a fusogenic protein (VSV‐G) to our existing E‐NoMi‐Red EV model to generate EVVs. (B) E‐NoMi‐Red EVVs do not require transfection agents for efficient cargo delivery. Fluorescent microscopy images of Cre‐reporter cells treated with E‐NoMi‐Red vesicles without (EVs—left) or with (EVVs—right) VSV‐G. E‐NoMi‐Red vesicles were loaded with Cre‐specific Nb (construct v_1.0_). Far‐red fluorescent (iRFP^POS^) cells representing the ON STATE of the Cre‐reporter were exclusively retrieved in the EVV condition and not in the EV condition. (C) E‐NoMi‐Red EVVs are more efficient than EVs to deliver Nb‐fused cargo. The bar plot represents the mean with SD (error bars) derived from three independent experiments. Conditions are the same as in (B), and flow cytometry read‐out is the number of iRFP^POS^ Cre‐reporter cells. (D) E‐NoMi‐Red EVVs isolated with immunocapture are more effective than those isolated with SEC. Fluorescent images of Cre‐reporter cells that were exposed to different Cre‐loaded EVVs isolated with immunocapture (bottom) or SEC (top). EVVs were isolated from E‐NoMi‐Red conditioned media, and loading occurred through Cre‐specific Nb (construct v_1.0_). (E) Flow cytometry assessment of (D). Percentage of iRFP^POS^ Cre‐reporter cells with immunocapture or SEC isolated E‐NoMi‐Red EVVs. The XY plot represents the mean with SD (error bars) derived from three independent experiments. (F) E‐NoMi‐Red EVVs are only effective with mCherry‐specific Nb‐based loading. Flow cytometry assessment of iRFP^POS^ Cre‐reporter cells postexposure to immunocaptured E‐NoMi‐Red EVVs loaded with Cre‐anti‐mCherry Nb (construct v_1.0_) compared to Cre‐nonspecific Nb (construct v_1.1_). (G) Representative image of next‐generation sequencing results. SaCas9 was fused to anti‐mCherry Nb (construct v_2.0_), loaded into E‐NoMi‐Red EVVs and isolated by immunocapture. Images are generated using the CRISPResso2 software and are representative images of next‐generating sequencing results of cells expressing sgRNA targeting *EMX1* post‐treatment with sham or loaded SaCas9 E‐NoMi‐Red EVVs. (H) Dose–response assessment of SaCas9‐loaded E‐NoMi‐Red EVVs. Increasing doses of E‐NoMi‐Red EVVs (0.5 × 10^6^–5.0 × 10^6^ EVVs/cell as determined by NTA) were evaluated based on their editing potential around the sgRNA targeting site of EMX1. Data was normalized to pDNA (encoding SaCas9‐Nb) transfection control. (I) Isolation‐based assessment of SaCas9‐loaded E‐NoMi‐Red EVVs coloaded with sgRNA. Equivalent doses of E‐NoMi‐Red EVVs (1.0 × 10^6^ EVVs/cell as determined by NTA), isolated by either immunocapture or SEC, were evaluated based on sgRNA targeting site of EMX1, compared to SaCas9‐loaded E‐NoMi‐Red EVs that were not coloaded with sgRNA. (Statistics) The data presented in this figure are depicted as the mean ± SD (error bars) obtained from normalized data across three independent replicates. Statistical analysis was performed using an unpaired *t* test (in C and F) or two‐way ANOVA (in E and I) and GraphPad Prism 10.2.1 software. *p < 0.05 and ***p < 0.001. ANOVA, analysis of variance; EV, extracellular vesicle; EVV, EV‐based vector; Nb, nanobody; NTA, nanoparticle tracking analysis; SD, standard deviation; SEC, size‐exclusion chromatography; VSV‐G, vesicular stomatitis virus G.

We next explored whether loading and enriching EVs with functional cargo is sufficient for effective delivery, without the use of transfection reagents. In previous experiments, we demonstrated the successful loading of EVs by transfecting producer cells with Lipofectamine and E‐NoMi‐Red Cre. We next compared Nb loading and delivery of E‐NoMi‐Red EVs and E‐NoMi‐Red EVVs without transfection agents using Cre‐reporter cells (Figure 4a). E‐NoMi‐Red EVVs are generated by incorporating VSV‐G. As previously reported, E‐NoMi‐Red EVs (VSV‐G^NEG^ vesicles) generated 4.0 ± 0.8 iRFP^POS^ reporter cells with lipofectamine, with a dose as low as 1.1 × 10^4^ particles/cell. However, without transfection agents, even exposure to ∼45‐times higher dose (i.e., 5.0 × 10^5^ particles/cell) was not able to induce any detectable iRFP signal in reporter cells (Figure 4b and Figure S3a). In contrast, 0.5 × 10^6^ E‐NoMi‐Red EVVs (VSV‐G^POS^ vesicles)/cell resulted in an iRFP signal in 78.1% ± 0.01% of our reporter cells with that same dose (Figure 4c). We also tested whether immunocapture enrichment was beneficial for Cre‐loaded E‐NoMi‐Red EVVs (VSV‐G^POS^) compared to standard SEC isolation. Conditioned media of cells expressing anti‐mCherry Nb tethered Cre, VSV‐G and E‐NoMi‐Red were divided and used to generate a dilution series (1.4 × 10^4^–1.1 × 10^6^ EVVs/cell estimated based on NTA readings) of immunocaptured and SEC‐isolated E‐NoMi‐Red EVVs (VSV‐G^POS^). After Cre‐reporter cells were exposed to EVVs, the immunocapture method needed 10‐times fewer particles/cell than the SEC method to achieve >80% of the cells, expressing iRFP fluorescence. Specifically, 1.2 × 10^5^ particles/cell generated 83.1% and 3.55% iRFP^POS^ reporter cells for immunocapture and SEC, respectively. (Figure 4d, e), suggesting that a larger percentage of vesicles are Cre‐loaded ENoMi‐Red‐EVVs when utilizing the immunocapture method compared to SEC. The difference between the immunocapture and SEC isolation method was confirmed with ‘ON’ and ‘OFF’ STATE Cre‐reporter RT‐qPCR (Figure S3b). Interestingly, the ‘ON’ STATE amplicon, resulting from the floxed reporter due to Cre activity, reached a plateau at 1.2 × 10^5^ particles/cell. Higher doses did not increase the detection of ‘ON’ STATE but did decrease the ‘OFF’ STATE. Indeed, 3.7 × 10^5^ and 1.1 × 10^6^ particles/cell significantly decreased the ‘OFF’ STATE in the immunocapture condition compared to the SEC condition (mean difference of 5.353 and 7.544 Cts, *p* < 0.01 and *p* < 0.05, respectively). This indicates that higher Cre activity/cell resulted in more effective Cre‐reporter switching from BFP to iRFP. Of note, for the experiments described above, HEK293T‐derived EVs/EVVs were exposed to HEK293T Cre‐reporter cells. To avoid transferring transfection agents from EV/EVV producer to EV/EVV recipient cells during our immunocapture isolation method, we exposed HeLa cells expressing our Cre‐reporter to the EVVs (Figure S3c). In contrast to HEK293T cells, HeLa cells are significantly less susceptible to Lipofectamine, the transfection agent used to express VSV‐G and Nb‐Cre in the EVV‐producer cells (Figure S3d). We observed activation of our Cre‐reporter in our HeLa Cre‐reporter cell line with immunocapture enriched EVVs. However, we needed 26‐times more particles/cell compared to HEK293T cells to generate at least 3% iRFP^POS^ cells (i.e., 3.7 × 10^5^ and 1.4 × 10^4^ particles/cell for HeLa and HEK293T, respectively). This highlights the difficulty of delivering functional payloads into HeLa cells compared to HEK293T cells. In addition to the merit of immunocapture compared to SEC, we also tested the advantage of E‐NoMi‐Red targeted loading with EVVs. We exposed Cre‐reporter cells to 0.5 × 10^6^ E‐NoMi‐Red EVVs (VSV‐G^POS^ vesicles) that were generated by cells expressing Cre coupled to an anti‐mCherry Nb or a nonspecific Nb (Figure 4f). E‐NoMi‐Red EVVs induced significantly more iRFP ^POS^ cells with the anti‐mCherry Nb than the nonspecific Nb (mean difference of 75.06 ± 15.4%, p < 0.01). The high recombination efficiency due to efficient loading by anti‐mCherry Nb in EVVs was confirmed with RT‐qPCR (Figure S3e). To further evaluate whether the E‐NoMi platform would be able to package larger cargos, EVVs were loaded with SaCas9 (Kleinstiver et al. 2015), a genome editing nuclease of 140 kDa (three times bigger than Cre recombinase). Importantly, E‐NoMi‐Red EVVs with SaCas9 (Kleinstiver et al. 2015) fused to a mCherry‐specific Nb fusion protein (construct v_2.0_) were incubated in cells expressing the sgRNA against the target gene *EMX1*, and modified reads due to gene editing activity at the expected cleavage site were detected in sgRNA‐expressing HEK293T cells (Figure 4g). Additionally, increasing doses of E‐NoMi‐Red EVVs (0.5 × 10^6^–5.0 × 10^6^ particles/cell as determined by NTA lead to an increasing % of indels detected (Figure 4h), suggesting a dose‐dependent effect in the delivery of E‐NoMi‐Red EVVs carrying SaCas9. To evaluate if our E‐NoMi‐Red EVVs could be co‐loaded with sgRNA, our producer cells were transfected to overexpress the sgRNA against *EMX1* in addition to the loading of SaCas9. After HEK293T cells were exposed to the same dose of E‐NoMi‐Red EVs coloaded with SaCas9 and sgRNA, the immunocapture method saw 10‐times increased editing efficiency when compared to the SEC method, with immunocapture and SEC methods yielding 5.68% and 0.53% editing efficiency, respectively. (Figure 4i). These EVVs were compared to immunocaptured EVVs which were not co‐loaded with sgRNA and only achieved 0.19 ± 0.2% editing efficiency. In conclusion, the E‐NoMi‐Red construct can be used to deliver functional protein cargo with different sizes and functions without the need for transfection reagents by incorporating fusogenic proteins on the surface of the EV/EVV.

### 3.5 E‐‘no'Mi‐Red EVs/EVVs Delivery After Removal of the Surface Enrichment Handle

**FIGURE 5:**
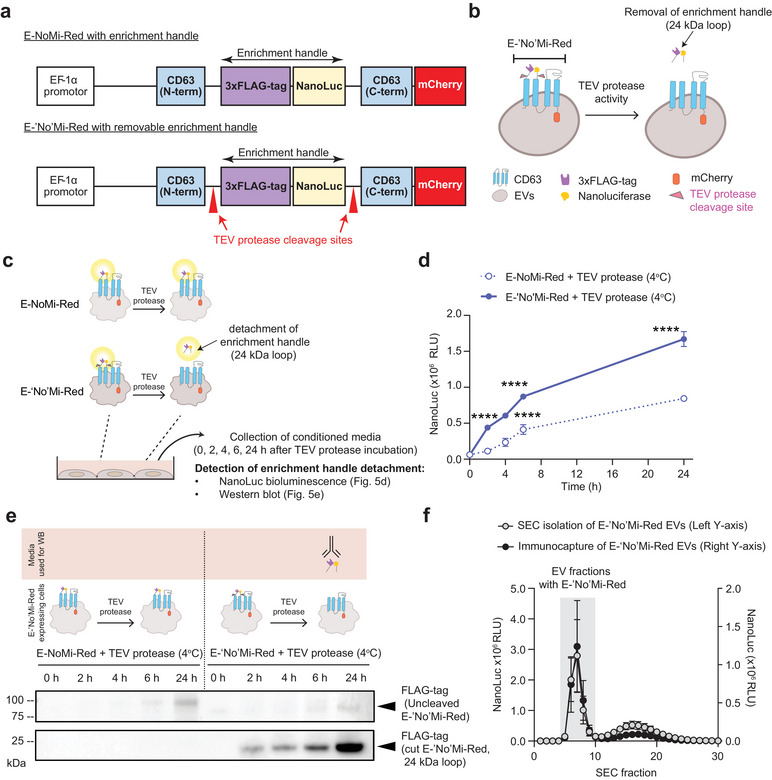
Preserving immunocapture potential with removable enrichment handle in ‘No'Mi‐Red EVs/EVVs. (A) E‐‘No'Mi‐Red construct with removable enrichment handle. The top cartoon illustrates the E‐NoMi‐Red, and the bottom cartoon shows the new E‐‘No'Mi‐Red construct. The term E‐‘No'Mi‐Red is noted with ' representing the position of the two TEV protease cleavage sites flanking the 3xFLAG‐tag and NanoLuc‐encoded sequence. (B, C) NanoLuc bioluminescence or FLAG‐tag detection can assess enrichment handle removal. Illustration describing the removal of 3xFLAG‐tag and NanoLuc from E‐‘No'Mi‐Red with TEV protease. TEV protease cleavage sites are sensitive to TEV protease activity, allowing for releasing a 24 kDa handle from the surface of our E‐‘No'Mi‐Red EVs. In (B), we illustrate the detachment of the handle from EVs, while in (C), we illustrate the detachment in living E‐‘No'Mi‐Red‐expressing cells. (D) Fast detachment of NanoLuc of E‐‘No'Mi‐Red post‐TEV protease treatment. E‐‘No'Mi‐Red‐expressing cell cultures were exposed to TEV protease at 4°C. Bioluminescence from NanoLuc, representing the enrichment handle, in extracted medium was measured at multiple time points (0, 2, 4, 6 and 24 h). E‐NoMi (without TEV sites) and E‐‘No'Mi (with TEV sites) samples were compared. (E) Tracking enrichment handle detachment with 3xFLAG‐tag detection. The extracted medium from the cell cultures in (D) was assessed by western blotting and anti‐FLAG‐tag. Immunoblot showed a 24 kDa enrichment handle in the TEV+ sample (bottom right), but not in the TEV‐ sample (bottom left). We also observed a gradual increase in the detection of FLAG‐tag at 78 kDa in the TEV‐ sample (top left), which is the predicted size of the uncleaved E‐‘No'Mi‐Red/E‐NoMi‐Red protein in EVs, indicating EV release with E‐NoMi‐Red. (F) TEV cleavage sites in our new construct do not compromise E‐‘No'Mi‐Red incorporation into EVs. E‐‘No'Mi‐Red EVs post‐isolation with immunocapture or SEC were applied to SEC. A peak of NanoLuc signal was observed in both samples in the SEC EV fractions, while the later SEC fractions, representing smaller non‐EV particulates, displayed minimal signal. (Statistics) The data presented in this figure are depicted as the mean ± SD (error bars) obtained from normalized data across three independent replicates. Statistical analysis was performed using two‐way ANOVA and GraphPad Prism 10.2.1 software. ****p < 0.0001. ANOVA, analysis of variance; EV, extracellular vesicle; EVV, EV‐based vector; Nb, nanobody; SD, standard deviation; SEC, size‐exclusion chromatography; TEV, tobacco etch virus.

**FIGURE 6:**
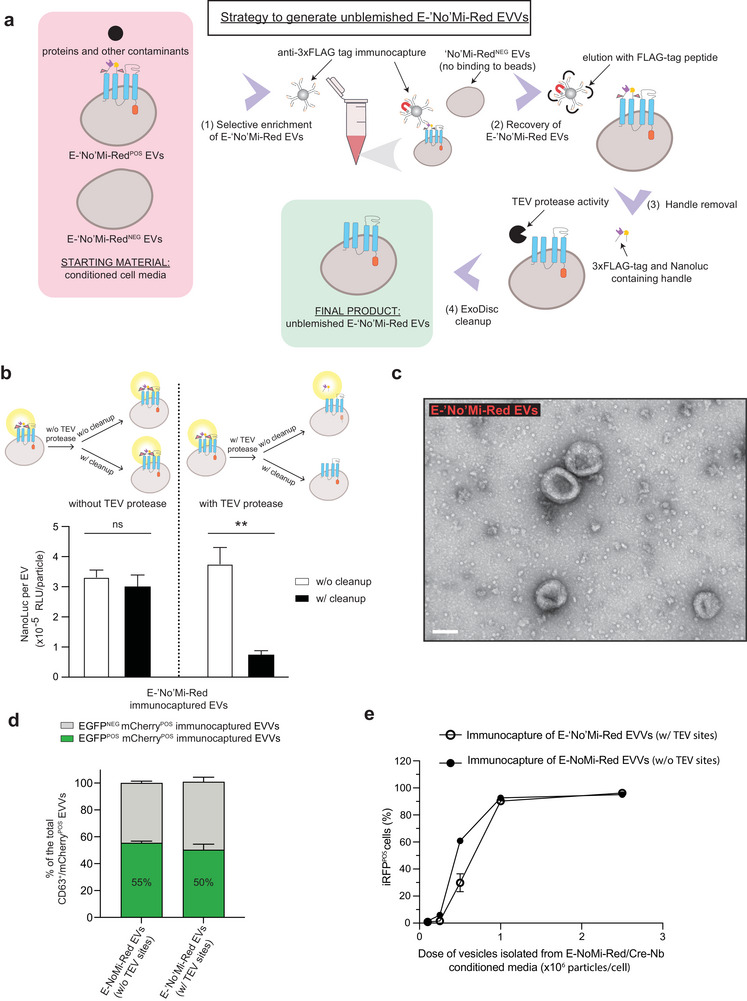
Clean‐up of TEV protease‐treated immunocaptured EVs to generate unblemished E‐‘No'Mi‐Red EVs. (A) Unblemished E‐‘No'Mi‐Red EV operation. Cartoon illustrating the operation to generate unblemished E‐‘No'Mi‐Red EVs through the cleanup of 3xFLAG‐tag and NanoLuc‐containing enrichment handle with the use of ExoDisc to purify EV sample from contaminants such as the enrichment handle and recombinant TEV protease. (B) Cleanup of enrichment handle postimmunocapture of E‐‘No'Mi‐Red EVs and TEV protease treatment. Data represents NanoLuc signal per EV (RLU/particle). Data on the left of the dotted line shows no significant changes in NanoLuc per EV levels, regardless of whether ExoDisc clean‐up was performed. Data on the right of the dotted line illustrates that when TEV protease is added, a significant drop in NanoLuc per EV levels is observed before and after clean‐up, showing both effective cleavage and effective cleanup of the EV sample. The top illustrates a cartoon of how experiments were performed. (C) Representative TEM images of immunocaptured E‐‘No'Mi‐Red EVs after TEV‐mediated cleavage of cargo handle and subsequent cleanup shows typical EV morphology. (D) Cargo loading is not significantly altered with anti‐mCherry Nb‐fused cargo in E‐‘No'Mi‐Red^POS^ EVs after TEV‐mediated cleavage of cargo handle. EVs were analysed for green fluorescence (EGFP^POS^) to compare loading efficiency of anti‐mCherry Nb EGFP compared to E‐NoMi‐Red EVs. The bars represent the percentage of CD63^POS^mCherry^POS^EGFP^POS^ EVs derived from three independent ExoView chips. (E) Flow cytometry assessment of EVV delivery after TEV‐mediated cleavage to assess whether cleavage and cleanup cause decreased delivery efficacy. Percentage of iRFP^POS^ Cre‐reporter cells with immunocapture E‐NoMi‐Red EVVs versus E‐‘No'Mi‐Red EVVs. (Statistics) The data presented in this figure are depicted as the mean ± SD (error bars) obtained from normalized data across three independent replicates. Statistical analysis was performed using a one‐way ANOVA and GraphPad Prism 10.2.1 software. **p < 0.01. ANOVA, analysis of variance; EV, extracellular vesicle; EVV, EV‐based vector; Nb, nanobody; SD, standard deviation; TEV, tobacco etch virus.

The cargo‐loaded EVs/EVVs immunocapture isolation method uses the 3xFLAG‐tag enrichment handle on the E‐NoMi‐Red EV surface to distinguish them from non‐NoMi‐Red EVs and free proteins. However, for future clinical applications, it might be desirable to remove this handle and maintain the native EV/EVV surface. Therefore, to develop ‘pristine’ EVs/EVVs, we genetically introduced two TEV protease cleavage sites flanking the 3xFLAG‐tag and NanoLuc‐encoding sequences of E‐NoMi‐Red (Figure 5a). We named this new construct E‐‘No'Mi‐Red, with ‘No’ representing the position of the cleavage sites sensitive to TEV protease activity that would allow the release of the 24 kDa handle from the surface of the EVs and creating an unblemished product (Figure 5b). We tested E‐‘No'Mi‐Red expressing cells to determine the time needed for TEV protease to detach the enrichment handle effectively (Figure 5c). The release of NanoLuc, a component of the enrichment handle, was assessed at different time points (0, 2, 4, 6 and 24 h) after TEV protease exposure in cell media. After 2 h, a higher NanoLuc signal was observed in the media (*p* < 0.0001) for both 4 and 37°C incubation conditions (Figure 5d and Figure S4a, respectively) in our samples with TEV sites (E‐‘No'Mi) compared to the samples without TEV sites (E‐NoMi). Western blot analysis of samples probed with an anti‐antibody confirmed that this increase in NanoLuc signal in the cell secretome was attributed to the detachment of the 24 kDa handle in the E‐‘No'Mi sample (Figure 5e and Figure S4b, c). In the E‐NoMi sample, we did not detect the 24 kDa handle but observed a gradual increase in the detection of FLAG‐tag at 78 kDa, which is the predicted size of the uncleaved E‐NoMi‐Red protein and could be due to E‐NoMi‐Red EV secretion. We explored whether incorporating TEV sites in the enrichment handle would compromise EV integrity or isolation with immunocapture. When applying conditioned E‐‘No'Mi‐Red cell media to the SEC, it resulted in a NanoLuc profile similar to the original E‐NoMi‐Red construct lacking the TEV sites in Figure 2a (Figure 5f). The peak of the NanoLuc signal in the SEC EV fractions indicated that when EVs were secreted from E‐‘No'Mi‐Red cells, the enrichment handle was still attached to the surface of the EVs. Indeed, EV extraction from the conditioned media with immunocapture was still feasible, and the SEC profile remained the same, confirming that the surface‐exposed handle remained intact even with the inclusion of TEV sites. We then tried to extract the enrichment handle from an immunocaptured E‐‘No'Mi‐Red EV sample by TEV protease treatment followed by ExoDisc cleanup to ensure an unblemished EV product (Woo et al. 2017) (Figure 6a). This system was implemented as a noninvasive tangential flow filtration cleanup to remove the handle and the TEV protease from our solution while larger EVs are retained. First, we assessed handle extraction through NanoLuc bioluminescence (Figure 6b). Immunocaptured E‐‘No'Mi‐Red EVs were exposed to TEV protease and split into two samples. Post‐ExoDisc cleanup, the NanoLuc signal per EV (based on bioluminescence and NTA readings) was decreased compared to without clean‐up. As a control condition, we tracked the NanoLuc signal of E‐‘No'Mi‐Red EV samples without TEV‐protease treatment, and they were not affected by the ExoDisc cleanup. We confirmed enrichment handle detachment of E‐‘No'Mi‐Red EVs post‐TEV protease activity and successful cleanup by western blots (Figure S5a, b). The enrichment handle was detectable with an anti‐Flag‐tag antibody at 24 kDa in our E‐‘No'Mi‐Red EV sample after TEV protease treatment. However, after ExoDisc cleanup, the 24 kDa fragment was absent in the E‐‘No'Mi‐Red EV sample after TEV protease treatment. However, as expected with an additional purification step, there is a slight loss of sample involved with the cleanup method that was observed across three replicate blots. As controls, we exposed E‐NoMi‐Red without TEV sites to TEV protease. As expected, it generated a full‐length construct at 75–150 kDa instead of the 24 kDa handle independent of TEV protease activity or ExoDisc cleanup. In all samples, we confirmed EV loading with the CD81 tetraspanin. TEM analysis was conducted after TEV protease treatment, which showed typical EV morphology after cleavage and cleanup (Figure 6c). ExoView analysis of cleaved E‐‘No'Mi‐Red EVs showed that loading was not disrupted and 50.2% ± 4.2% loading was still achieved (Figure 6d) compared to loading of E‐NoMi‐Red, which as reported was 55.3% ± 1.5%. To ensure functionality of our EVVs after TEV‐protease cleavage, increasing doses of cleaved EVVs (0.1 × 10^6^–2.5 × 10^6^) were added to Cre‐reporter cells and showed minimal changes to delivery efficacy (Figure 6e). Here, we show that it is possible to enrich for cargo‐containing EVs by immunocapture without exposing potential antigenic peptides on the surface of EVs in the final isolation.

### 3.6 E‐‘No'Mi‐Red EVV‐Mediated Delivery of Cre Into Xenograft and Glioblastoma Bearing Mouse Models

**FIGURE 7:**
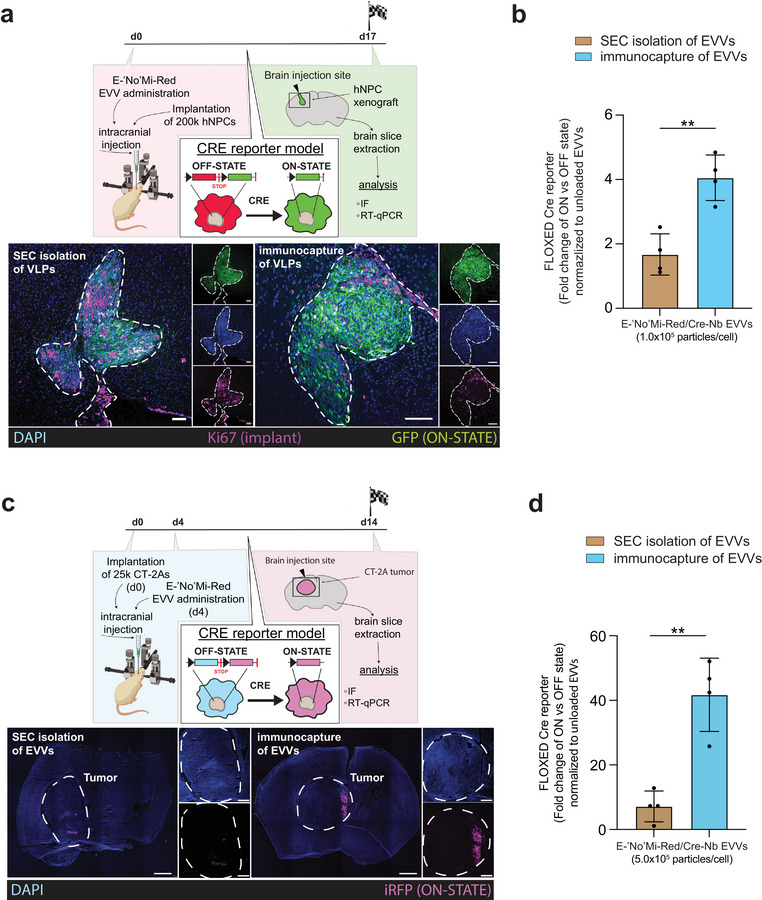
E‐‘No'Mi‐Red EVVs in in vivo brain implantation models. (A) Outline of hNPC xenograft model for intracranial E‐‘No'Mi‐Red EVV administration into the brain. hNPCs express a Cre‐reporter that changes from red to green fluorescence upon Cre activity. Cells are coimplanted intracranially with the E‐‘No'Mi Red EVVs (without cargo isolated with immunocapture, with cargo: isolated with SEC or isolated with immunocapture). Analysis by immunofluorescence (IF) and RT‐qPCR of brain‐implanted hNPCs occurred on Day 17. Confocal microscopy images of hNPC engraftment site. Brain sections containing the hNPCs of interest were identified by anti‐Ki67 staining and analysed for ON STATE Cre‐reporter activity (GFP). DAPI labelled both the mouse brain and hNPC nuclei. Scale bars represent 100 µm. The image is representative of three replicates. (B) Cre‐activity posttreatment of implanted hNPCs with E‐‘No'Mi Red EVVs. Brain sections were used to extract RNA of implanted hNPCs and perform RT‐qPCR assessment of Cre activity with primers distinguishing the ON and OFF STATE of the hNPC‐encoded Cre‐reporter. Data represents the fold change of ON and OFF STATE Cts, normalized to the unloaded EVV control. (C) Outline of CT‐2A tumour model for intracranial E‐‘No'Mi‐Red EVV administration into the brain. CT‐2A cells express a Cre‐reporter that changes from BFP to iRFP fluorescence upon Cre activity. Cells were implanted intracranially and treated 4 days later at the injection site with the E‐‘No'Mi Red EVVs (unloaded isolated with immunocapture, with cargo: isolated with SEC or isolated with immunocapture). Analysis by IF and RT‐qPCR of brain‐implanted cells occurred on Day 14. Confocal microscopy of the whole brain with CT‐2A tumour (circled) treated with Cre‐loaded E‐‘No'Mi Red EVVs. Brain sections treated with E‐‘No'Mi Red EVVs posttumour implantation were assessed based on fluorescence. The ON‐STATE of the Cre‐reporter was visualized with far‐red (iRFP) fluorescence. DAPI staining was also performed to illustrate the mouse brain and tumour cells. Scale bars represent 1000 µm in the left panel and 500 µm in the right panels. The images are representative of two replicates per group. (D) Cre‐activity posttreatment of implanted CT‐2A tumour cells with E‐‘No'Mi Red EVVs. Brain digests of dissected tumours were used to isolate a single cell suspension of tumour cells to extract RNA of implanted CT‐2As and perform RT‐qPCR assessment of Cre activity with primers distinguishing the ON and OFF STATE of the Cre‐reporter. Data represents the fold change of ON and OFF STATE Cts, normalized to the unloaded EVV control. (Statistics) The data presented in this figure are depicted as the mean ± SD (error bars) obtained from normalized data. Statistical analysis was performed using an unpaired *t* test and GraphPad Prism 10.2.1 software. **p < 0.01, ***p < 0.001 and ****p < 0.0001. EV, extracellular vesicle; EVV, EV‐based vector; hNPC, human neuronal precursor cell; SD, standard deviation; SEC, size‐exclusion chromatography.

To assess the suitability of our delivery platform for in vivo applications, we conducted experiments using two different mouse models. A xenograft model was explored where human neuronal precursor cells (hNPCs) and EVVs were coimplanted in the brains of 21 immunocompromised mice as a cargo delivery model with human cells (Maalouf et al. 2023). The hNPCs stably expressed a lentiviral construct with a Cre recombinase inducible dsRed to GFP cassette (Figure 7a). Seventeen days after hNPC (2.0 × 10^5^ cells) implantation and the simultaneous injection of E‐‘No'Mi‐Red EVVs (1.0 × 10^5^ EVVs/cell), mice were sacrificed and brain sections were analysed for Cre activity using fluorescent microscopy (three mice per group) or RT‐qPCR (four mice per group). The GFP fluorescence is indicative of functional delivery of Cre to the reporter xenograft. Cre recombination was most prominent in brain sections treated with immunocaptured E‐‘No'Mi‐Red Cre containing EVVs compared to sections exposed to either SEC isolated E‐‘No'Mi‐Red Cre containing EVVs or unloaded E‐‘No'Mi‐Red EVVs (Figure 7a and Figure S6a). Based on RT‐qPCR, we detected the highest recombination events in implants treated with immunocaptured E‐‘No'Mi‐Red EVVs (Figure 7b). Immunocaptured Cre‐loaded E‐‘No'Mi‐Red EVVs generated more Cre activity (4.1 ± 0.7 fold change, normalized to unloaded EVVs) in the hNPC‐implant than those isolated through SEC (1.7 ± 0.6 fold change, normalized to unloaded EVVs). We further evaluated whether the EVV‐mediated Cre delivery would be applicable in a brain tumour model with EVVs injected 4 days after tumour implantation (Figure 7c). CT‐2A glioma cells (2.0 × 10^4^) stably transduced with a BFP to iRFP Cre‐reporter and implanted into the left‐hemisphere of eighteen syngeneic mice. Four days later, E‐‘No'Mi‐Red EVVs (5.0 × 10^5^ EVVs/cell) in a second injection were administered to assess the delivery of our EVVs to a brain tumour model. Fluorescent microscopy (Figure 7c and Figure S6b) and RT‐qPCR (Figure 7d) were performed after 14 days. Remarkably, fluorescent microscopy (two mice per group) and RT‐qPCR (four mice per group) both demonstrated that immunocaptured E‐‘No'Mi‐Red EVVs loaded with Cre‐Nb exhibited significantly higher Cre activity (41.7 ± 11.3 fold change) compared to SEC isolated EVVs (7.2 ± 4.8 fold change), both normalized to the unloaded EVV group (Figure 7d). This five‐time delivery increase using the immunocapture method highlights the importance of sample enrichment for in vivo purposes. In conclusion, immunocapture VSV‐G‐containing vesicles generated with our E‐‘No'Mi‐Red outperform those isolated with a standard EV isolation method and can be used to deliver cargo to cells implanted in the brain.

## 4 Discussion

EVs are spontaneously released from cells in diverse sizes and shapes (Zabeo et al. 2017), carrying various cargo types (Kim et al. 2013) and biomolecules at different concentrations per vesicle (Willms et al. 2018). EVs heterogeneity makes them less attractive from a gene therapy perspective compared to carriers like viral vectors and liposomes, where one strives for a homogeneous solution with the largest EV proportion contributing to the therapy and maximum drug payload per vesicle to attain effective treatment in patients. Here, we tackled these issues by designing an EV platform compatible with different recombinant payloads with different cargo sizes, such as SaCas9 and Cre. Through transgene expression in the EV‐producer cells, we engineered a protein that projects handles on the outer and inner surfaces of EVs. The outer enrichment handle increases the purity of desired EVs to 90%, while the inner cargo handle increases EV loading by 13‐times compared to nontargeted loading. This improvement is attributed to the high affinity between the immunocapture beads and the enrichment handle, as well as between the Nb fused to a desired payload and the cargo handle. Using a noncovalent affinity system, EVs captured with the handle can be recovered from heterogenous solutions, and their recombinant functional payload is detached once uptake is made by the recipient cells. As demonstrated in reporter cell lines and animal models, using these engineered EVs resulted in more efficient cargo delivery for gene therapy, compared to standard EV preparations. Furthermore, we were able to remove the enrichment handle after immunocapture isolation to decrease potential immunogenicity or toxicity that might occur due to the presentation of antigens on the outer EV surface (Théry et al. 2009; Wang et al. 2022; Zhang et al. 2024). The incorporation of TEV sites, necessary for handle removal, did not compromise the immunocapture capabilities of these EVs. Our platform was validated with three types of payloads (i.e., EGFP, Cre recombinase and SaCas9) to assess EV‐loading and functionality in EV‐recipient cells both in vitro and in vivo. EGFP fluorescence and western blot analysis of isolated EVs indicated a preference for transferring recombinant protein with our method. By testing EVs with Cre‐reporter cells, we established that recombinant protein transfer occurred instead of nucleic acids transfer, the well‐established cargo type for traditional carriers such as viral vectors and synthetic particles (Li et al. 2022; Piperno et al. 2021). Cre fused to an Nb targeting our E‐NoMi construct demonstrated greater efficacy in EV‐mediated transfer compared to fusion with a nonspecific Nb. This underscores the importance of protein transfer, as Cre mRNA is overexpressed in both conditions and transcript production occurs in the EV‐producer cells (Rufino‐Ramos et al. 2023). The loading strategy used here also overcomes the inherent limitations of other, outdated loading strategies that rely on exogenous loading of vesicles. Exogenous methods such as chemical loading and electroporation introduce potentially cytotoxic residual agents to the EV samples and cause degradation and aggregation of EVs (Leandro et al. 2024; Rufino‐Ramos et al. 2017).

The high affinity to tags, easy recombinant production by expression bacteria and small size made Nbs particularly useful in the EV field. Previously, Nb binding to the exterior of purified EVs facilitated EV isolation, cell targeting, both in vitro and preclinical disease models (Kooijmans, Aleza, et al. 2016; Kooijmans et al. 2018a, Kooijmans et al. 2018b; Kooijmans et al. 2016; Pham et al. 2021; Scott et al. 2022; Xia et al. 2023) and was used for studying EV‐uptake by recipient cells (Joshi et al. 2020). Here, we leveraged the ability of mammalian cells to express Nbs and fused them with desired payloads. Expression of hypercompact Nbs in the producer cell eliminated the need to compromise EV membranes through chemical or physical (exogenous) loading methods to enrich EVs with a payload, like sonication, electroporation or pH alteration for incorporating Nbs (Colja et al. 2023; Keener 2020). Although genetic cargo transfer applications have been extensively explored, protein‐based gene therapy remains relatively unexplored. The lag in protein transfer modalities may stem from the drawbacks associated with protein delivery, including its tendency to occupy more space than RNA or DNA, thereby limiting cargo capacity, expensiveness and difficult production of larger proteins (Di Ianni et al. 2025). In addition, there is a lack of promoters that can regulate cell type or cell status expression and the temporary presence of proteins in cells compared to some mRNA or DNA delivery methods. However, protein transfer has advantages such as directly modulating cellular processes or signalling pathways, bypassing longsome gene expression and offering a more rapid and transient therapeutic response than conventional gene therapy. Shorter modulation is more suitable for certain applications that are prone to potential off‐target effects. This is further reinforced by advancements in CRISPR technologies, where precise gene modulation is desired only temporarily until the desired genetic changes are achieved to reduce off‐target effects (Stahl et al. 2023). Furthermore, this trend is anticipated to escalate with the emergence of the de novo enzyme design field (Notin et al. 2024).

Our approach aided in selecting payload‐rich EVs, and their cargo was proven to be functional in recipient cells. Nevertheless, we could not produce a strong signal in the recipient cells without using transfection agents with a limited number of EVs per cell. In this regard, our results are consistent with other studies that maximized EV payload, even with Nb‐based loading of EVs, which also reported only a marginal delivery in the target cells (Ye et al. 2020). We thus considered equipping our EVs with fusogenic proteins to facilitate delivery. Indeed, a fusogenic EV configuration was able to facilitate EV‐mediated protein delivery without transfection agents in a dose‐dependent manner, potentially due to the highly fusogenic properties of VSV‐G. Hereby, we emphasized the need for tools to enhance targeted delivery in addition to efficient loading and homogeneity of EV suspensions for efficient delivery of EV cargo.

The development of engineered EVs has garnered immense promise for clinical translation. However, translating this potential into clinical applications requires scalable and efficient production methods. The combination of immunocapture and Nb‐based targeted loading encourages the ability for engineered EVs to be produced in a scalable manner with bioreactor‐level production. Producer cells can be bioengineered to stably express the E‐NoMi‐Red cassette and passively produce engineered EVs. The stable expression of E‐NoMi‐Red ensures consistent product quality and simplifies the manufacturing process.

Coupled with the modularity of Nb‐fused cargo, this approach allows for the production of a wide range of engineered EVs with different therapeutic payloads, all from the same engineered cell line. This eliminates the need to establish and maintain multiple cell lines or switch out the existing cell line being cultured, both significant bottlenecks in large‐scale EV production. The final product being efficiently isolated without the need for complicated machinery or techniques also provides a seamless protocol that is quick and ensures a high quality of isolation.

Our system still has several limitations that need to be addressed in future iterations. Loading efficiency remains a challenge, with only about 55% of EVs successfully incorporating the cargo. Other nonimmunogenic protein‐Nb pairs should be investigated to find options with increased loading efficiency, while decreasing immunogenicity. Similarly, EV‐enriched proteins other than CD63 should be investigated as the scaffold for the cargo handle; other proteins such as CD81 or CD9 may provide additional loading or enrichment efficiency. Additionally, the ExoDisc cleanup process leads to sample loss, which can affect yield and reproducibility. Although VSV‐G is commonly used in many studies, it introduces potential immunogenicity concerns. Another key limitation is the lack of cell‐type targeting, which currently requires local injection, limiting the system's potential for broader clinical use.

In conclusion, we adopted the E‐NoMi construct previously used for diagnostic purposes (Maalouf et al. 2023) into a platform that enriches for EVs with an Nb‐fused cargo. This enrichment method generated a more homogenous EV preparation that was more effective in delivering cargo than EV preparations gained by standard methods, which do not remove unloaded EVs. Additionally, we showed the integrity and functionality of this platform by creating a ‘unblemished’ EV after removing the outer handle, essential for a selective enrichment of loaded EVs but dispensable for downstream targeting and therapeutic purposes.

## Author Contributions

W.O., A.Z.P. and K.B. performed experiments, analysed data, and wrote and edited the paper. E.D.I., D.M.F., K.M., L.N., K.L., T.X. and P.R. aided with in vivo experiments. C.A.V. and D.C.B. provided insight on the maintenance of hNPCs. D.R.R. and B.P.K. provided insight on genome editing, generated the gRNAs used and helped edit the paper. H.L., M.A.M. and X.O.B. provided guidance on the design and analysis of experiments and helped edit the paper. K.B. conceptualized the study, planned, and supervised experiments, and wrote and edited the paper.

## Conflicts of Interest

An invention disclosure (2022‐558) has been filed. Benjamin P. Kleinstiver is an inventor on patents or patent applications filed by MGB that describe genome engineering technologies. Benjamin P. Kleinstiver is a consultant for EcoR1 capital and Novartis Venture Fund and is on the scientific advisory boards of Acrigen Biosciences, Life Edit Therapeutics and Prime Medicine. Benjamin P. Kleinstiver has a financial interest in Prime Medicine, Inc., a company developing therapeutic CRISPR‐Cas technologies for gene editing. Benjamin P. Kleinstiver's interests were reviewed and are managed by MGH and MGB in accordance with their conflict‐of‐interest policies. The other authors declare no conflicts of interest.

## Supporting information



Supporting Information

## Data Availability

The authors confirm that the data supporting the findings are available within the article and its supplementary material. Raw data that support the findings reported in this study are available upon reasonable request.
