# Supplementary material for: Engineering of CD63 Enables Selective Extracellular Vesicle Cargo Loading and Enhanced Payload Delivery
Source: J Extracell Vesicles. 2025 Jun 17;14(6):e70094. doi: 10.1002/jev2.70094 (PMC12173531; doi:10.1002/jev2.70094)
Supplement: Supplementary file 1 — Supporting Information [file JEV2-14-e70094-s001.docx]

**SUPPLEMENTARY INFORMATION**

**
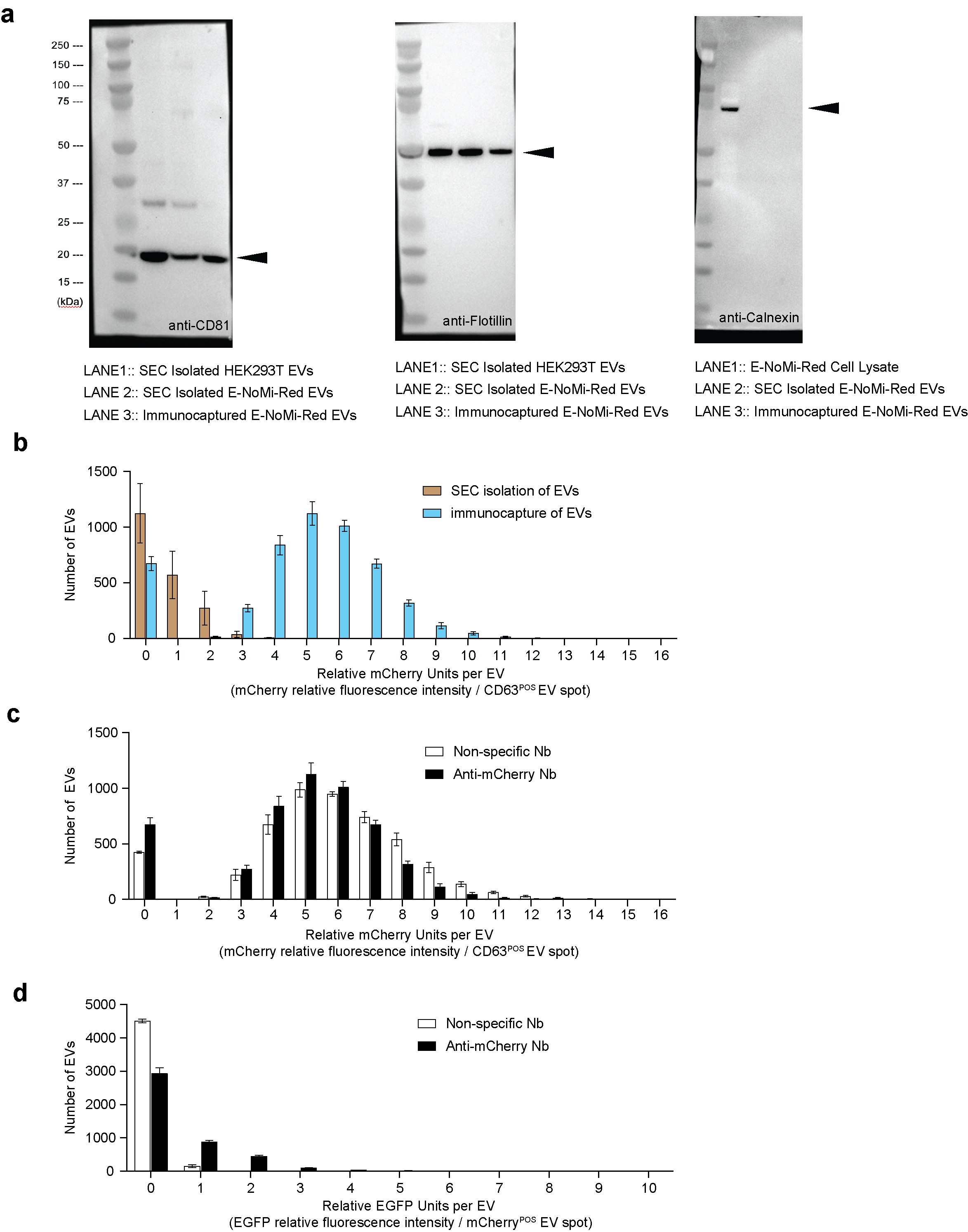
**

**Figure S1**

(A) Full blot of Figure 2 (e).

(B) Distribution of mCherry fluorescent intensity in a CD63-expressing EV solution. EV suspensions generated with SEC or immunocapture were assessed on the CD63^POS^ EV spots of an ExoView™ chip for red fluorescence. Bars represent the numbers of EVs captured by immobilized anti-CD63. Data were derived from single-EV counting of three independent spots on ExoView™ chip.

(C) E-NoMi-Red^POS^ EV enrichment is not compromised by endogenous Nb-fused cargo loading. EVs were isolated with immunocapture from conditioned media derived from E-NoMi-Red expressing cells expressing Nb-fused EGFP (non-specific and anti-mCherry Nb). Distribution of mCherry fluorescent intensity from CD63^POS^ EV spot of three independent spots was compared between cells expressing non-specific Nb (white bar) and anti-mCherry Nb (black bar).

(D) Cargo loading is improved with anti-mCherry Nb-fused cargo in E-NoMi-Red^POS^ enriched EVs. EVs assessed in Figure 2F were analyzed for green fluorescence (EGFP^POS^) to compare loading efficiencies between cargo fused to non-specific Nb or anti-mCherry Nb. The bars represent the number of CD63^POS^mCherry^POS^ EVs derived from three independent ExoView™ spots against relative EGFP fluorescent intensity.

**
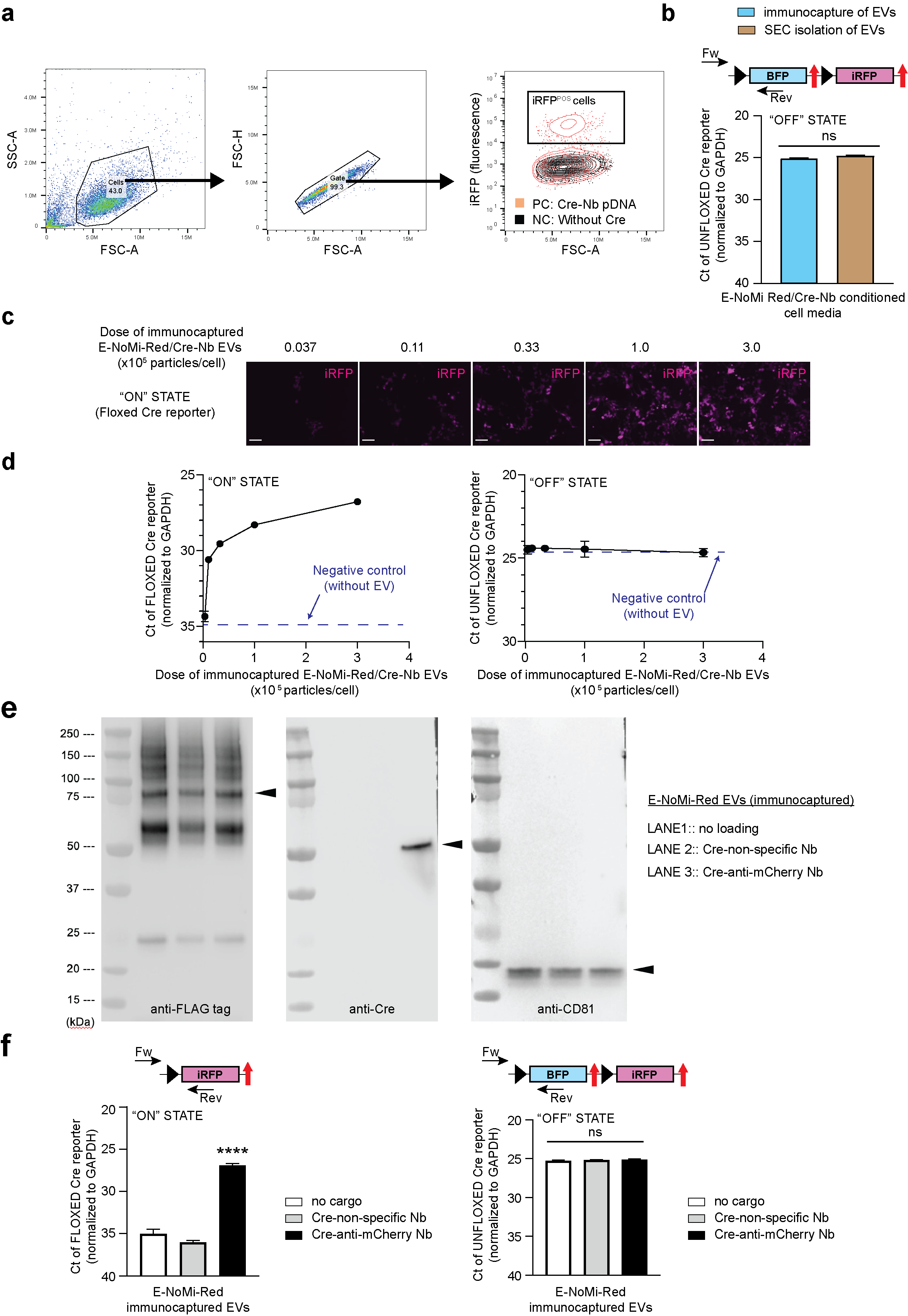
**

**Figure S2**

(A) Gating strategy for Cre-reporter cells used to select far-red positive cells post-EV-delivery of Cre. In black, the negative control (only reporter cells), and in red, the positive control (transfected with Cre plasmid) is shown. Analysis was performed with FlowJo software.

(B) Validation of observations in Figure 3 (B) and (C) on the genomic level. RT-qPCR of cDNA extracted from Cre-reporter cells transfected with Cre-loaded E-NoMi-Red EVs, showing no differences in “OFF” STATE Cre-reporter expression between immunocaptured (blue bar) compared to the SEC (brown bar) condition. PCR strategy is illustrated above the bar graphs, and the bar graphs represent the mean with SD (error bars) from three independent experiments.

(C) Dose-dependent response of cargo-loaded immunocaptured E-NoMi-Red EVs. The Cre-reporter cells were treated with increasing doses of Cre-loaded E-NoMi-Red EVs ranging from 3.7x103 to 3.0x105 particles/cell, and Cre-reporter cells were evaluated through fluorescent microscopy. The scale bar represents 100 µm.

(D) Validation of observations in Figure 3 (E) on the genomic level. RT-qPCR of cDNA extracted from Cre-reporter cells transfected with increasing doses of Cre-loaded E-NoMi-Red EVs. RT-qPCR quantified “ON” STATE and “OFF” STATE of the Cre-reporter. The XY plot represents the mean with SD (error bars) from three independent experiments.

(E) Full blot of Figure 3 (G).

(F) Validation of observations in Figure 3 (H). RT-qPCR of cDNA extracted from Cre-reporter cells transfected with E-NoMi-EVs not loaded (white), loaded through non-specific Nb (construct V1.1), or specific Nb (construct V1.0). RT-qPCR quantified “ON” STATE and “OFF” STATE of the Cre-reporter. PCR strategy is illustrated above the bar graphs, and the bar graphs represent the mean with SD (error bars) from three independent experiments.

(Statistics) Statistical analysis was performed using an unpaired t-test (in B) or one-way ANOVA (in F) and GraphPad Prism 10.2.1 software. ****p< 0.0001.


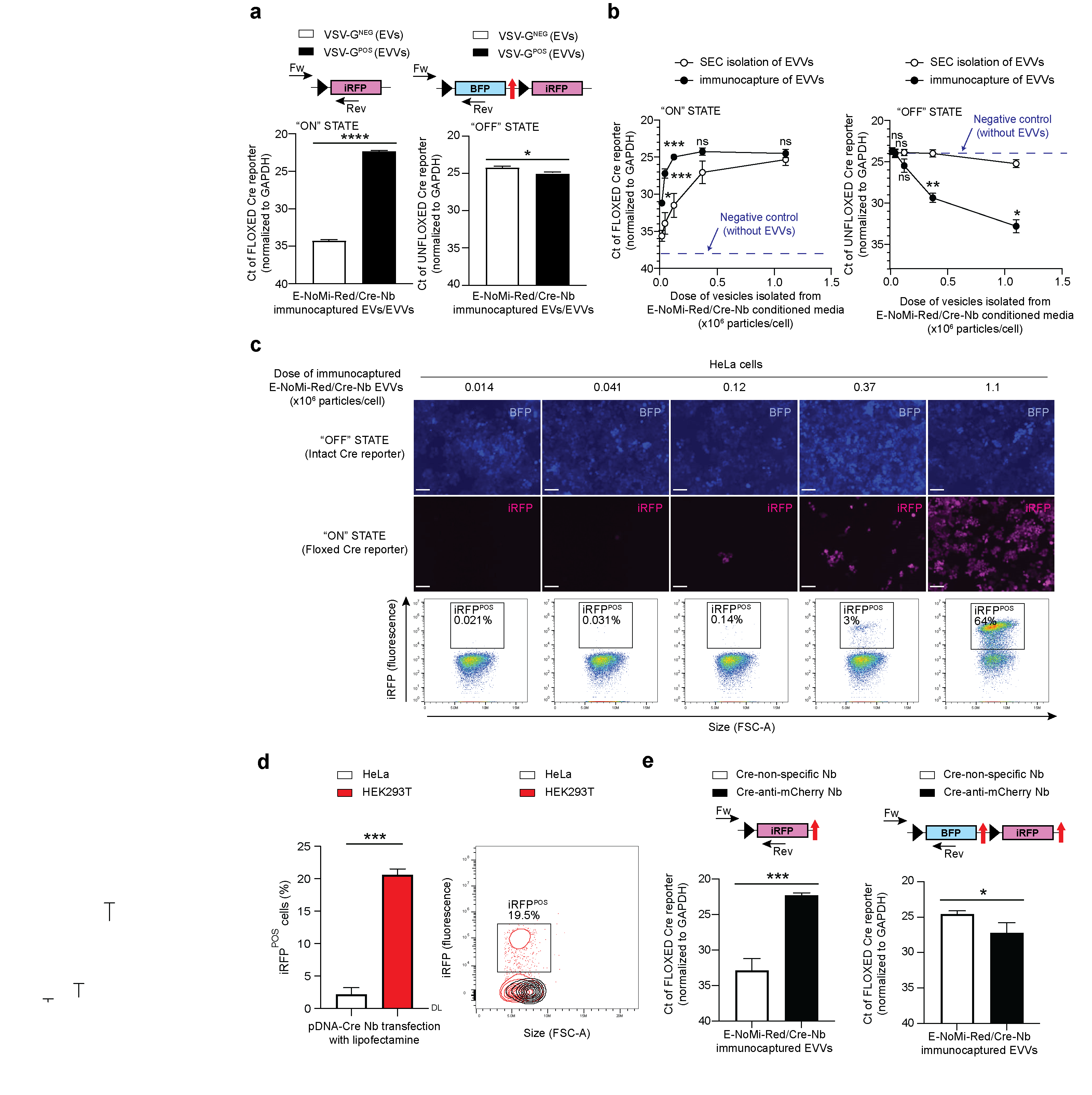


**Figure S3**

(A) Validation of observations in Figure 4 (B) and (C). RT-qPCR of cDNA extracted from Cre-reporter cells treated with E-NoMi-Red vesicles without (EVs - left) or with (EVVs - right) VSV-G. RT-qPCR quantified “ON” STATE and “OFF” STATE of the Cre-reporter. PCR strategy is illustrated above the bar graphs, and the bar graphs represent the mean with SD (error bars) from three independent experiments.

(B) Validation of observations in Figure 4 (D) and (E). RT-qPCR of cDNA extracted from Cre-reporter cells treated with different Cre-loaded EVVs isolated with immunocapture (closed symbol) or SEC (open symbol). RT-qPCR quantified “ON” STATE and “OFF” STATE of the Cre-reporter. The XY plot represents the mean with SD (error bars) from three independent experiments.

(C) Dose-dependent response of cargo-loaded immunocaptured E-NoMi-Red EVVs on HeLa cells. The Cre-reporter HeLa cells were treated with increasing doses of Cre-loaded E-NoMi-Red EVVs ranging from 1.4x10^4^ to 1.1x10^6^ particles/cell, and Cre-reporter cells were evaluated through fluorescent microscopy (top and middle). The scale bar represents 100 µm. Flow plots illustrate far-red fluorescent (iRFP^POS^) cells representing the ON STATE of the Cre-reporter in cells (bottom).

(D) HeLa cells are difficult to transfect with lipofectamine. pDNA expressing Cre-fused Nb was transfected into HeLa (white bar) or HEK293T (red bar) cells. Bar graphs represent the mean with SD (error bars) from three independent experiments.

(E) Validation of observations in Figure 4 (F). RT-qPCR of cDNA extracted from Cre-reporter cells transfected with immunocaptured E-NoMi-Red EVVs loaded through non-specific Nb (construct V1.1) or specific Nb (construct V1.0). RT-qPCR quantified “ON” STATE and “OFF” STATE of the Cre-reporter. PCR strategy is illustrated above the bar graphs. The bar graphs represent the mean from three independent experiments and each plot represents each independent value.

(Statistics) Statistical analysis was performed using an unpaired t-test (in A, D and E) or two-way ANOVA (in B) and GraphPad Prism 10.2.1 software. *p < 0.05, **p < 0.01, ***p< 0.001 and ****p< 0.0001.


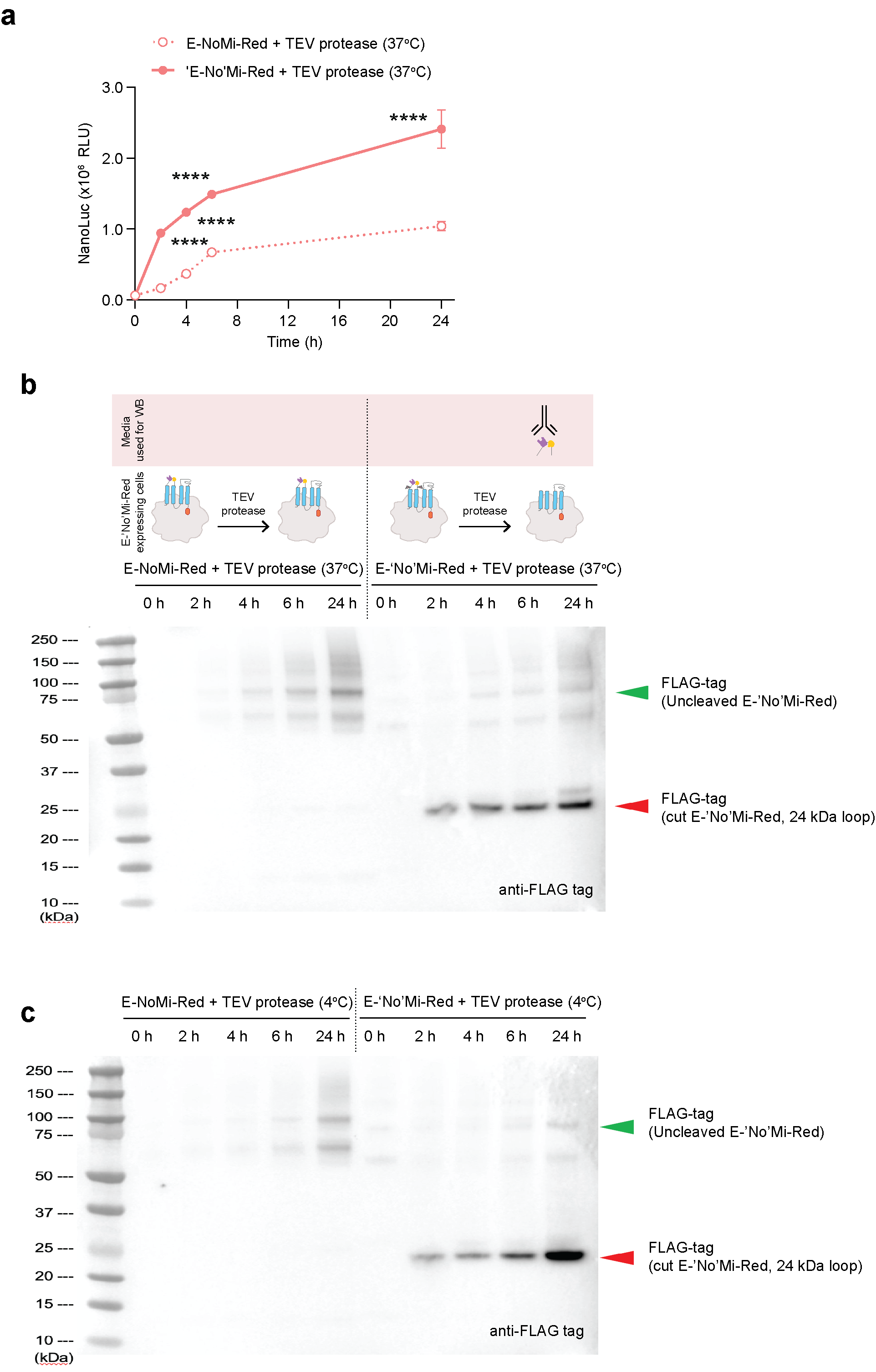


**Figure S4**

(A) Fast detachment of NanoLuc-containing enrichment handle of E-'No'Mi-Red post-TEV protease treatment. E-'No'Mi-Red-expressing cell cultures were exposed to TEV protease at 37°C. Bioluminescence derived from NanoLuc, representing the enrichment handle, in the extracted medium was measured at multiple time points (0h, 2h, 4h, 6h, 24h). E-NoMi (without TEV sites) and E-‘No’Mi (with TEV sites) samples were compared.

(B) Tracking enrichment handle detachment with FLAG-tag detection. The extracted medium from the cell cultures in Figure S4 (A) was tested with western blotting with anti-FLAG-tag antibody. Immunoblot showed a 24kDa enrichment handle in the TEV+ sample (right of the dotted line), but not in the TEV- sample (left of dotted line). We also observed a gradual increase in the detection of FLAG-tag at 78kDa in the TEV- sample, which is the predicted size of the uncleaved E-'No'Mi-Red/E-NoMi-Red protein in EVs. At 37°C, more EV production is expected by cells than at 4°C (Figure 5e), explaining the uncleaved E-NoMi-Red handle in the media.

(C) Full blot of Figure 5 (E).


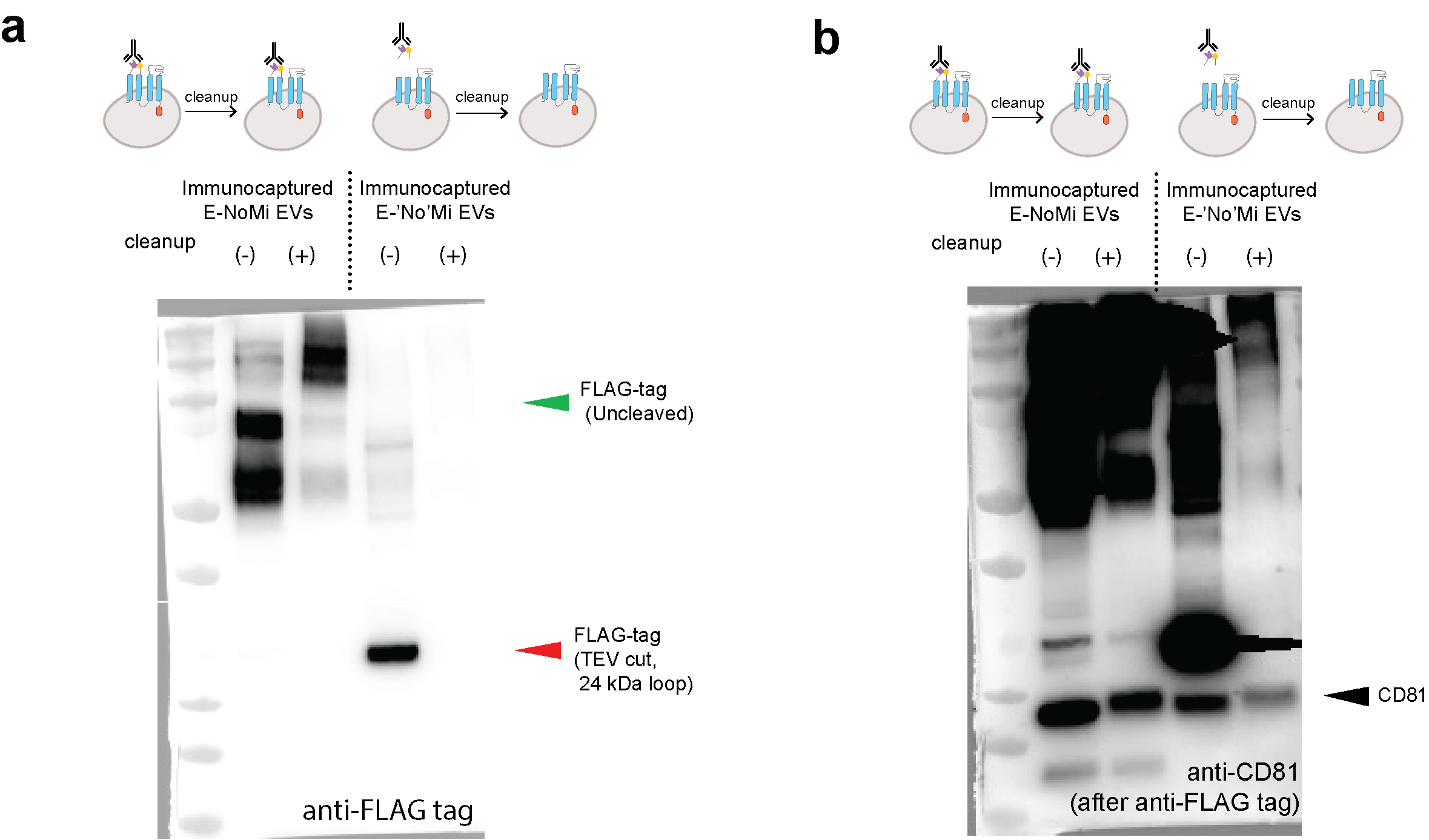


**Figure S5**

(A) Confirmation of **Figure 6b** through 3xFLAG-tag detection. Western blot showing E-NoMi-Red EVs (without TEV cleavage sites) and E-‘No’Mi-Red EVs (with TEV cleavage sites) treated with TEV protease. There is no detectable 24kDa handle band in either the E-NoMi-Red EV sample with(out) TEV (left of the dotted line). With the TEV cleavage sites in the E-‘No’Mi-Red EV sample (right of the dotted line), a detectable 24kDa band demonstrates efficient cleavage of the enrichment handle. However, when utilizing ExoDisc^TM^ cleanup, there is no FLAG-tag band at 24kDa.

(B) Verification of EV loading in each lane, even in cases where the handle is undetectable, we assessed the presence of anti-CD81 signal.


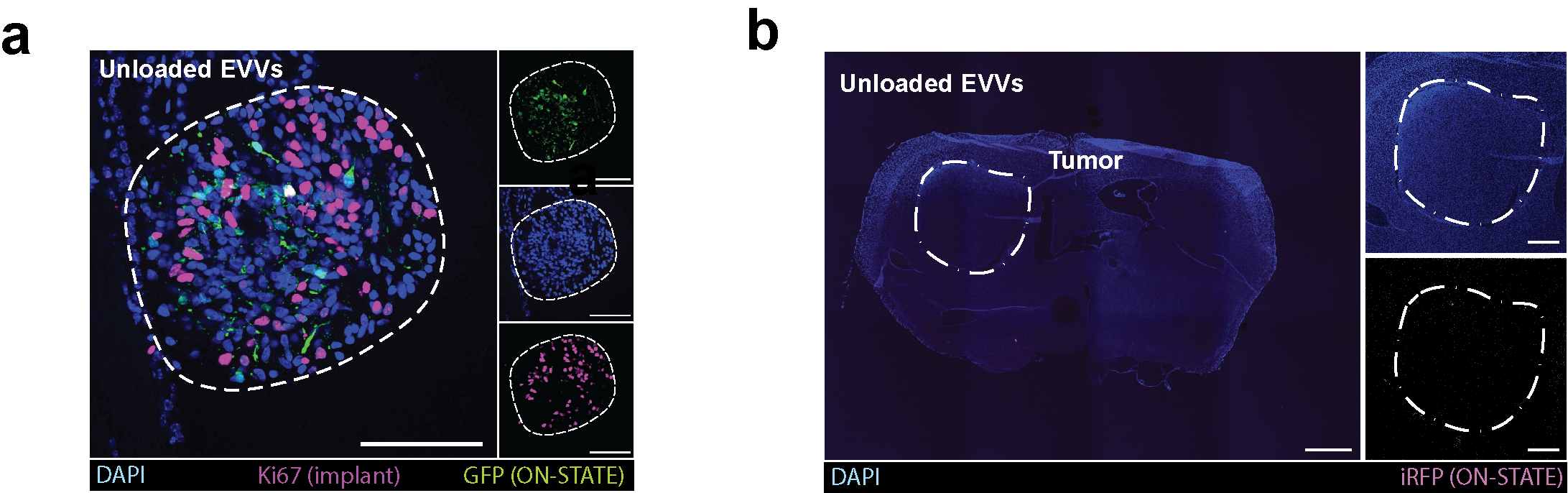


**Figure S6**

(A) Brain section for the unloaded E-‘No’Mi-Red EVVs control, the hNPCs of interest were identified by anti-Ki67 staining and analyzed for ON-state Cre-reporter activity (GFP). DAPI labeled both the mouse brain and hNPC nuclei. Scale bars represent 100 µm.

(B) Brain section for the unloaded E-‘No’Mi-Red EVVs control, the CT-2A tumor was identified via Dapi (BFP) signal and the ON-state Cre-reporter activity (iRFP). Scale bar on the left panel represents 1000 µm and the scale bars in the right panels represents 500 µm.


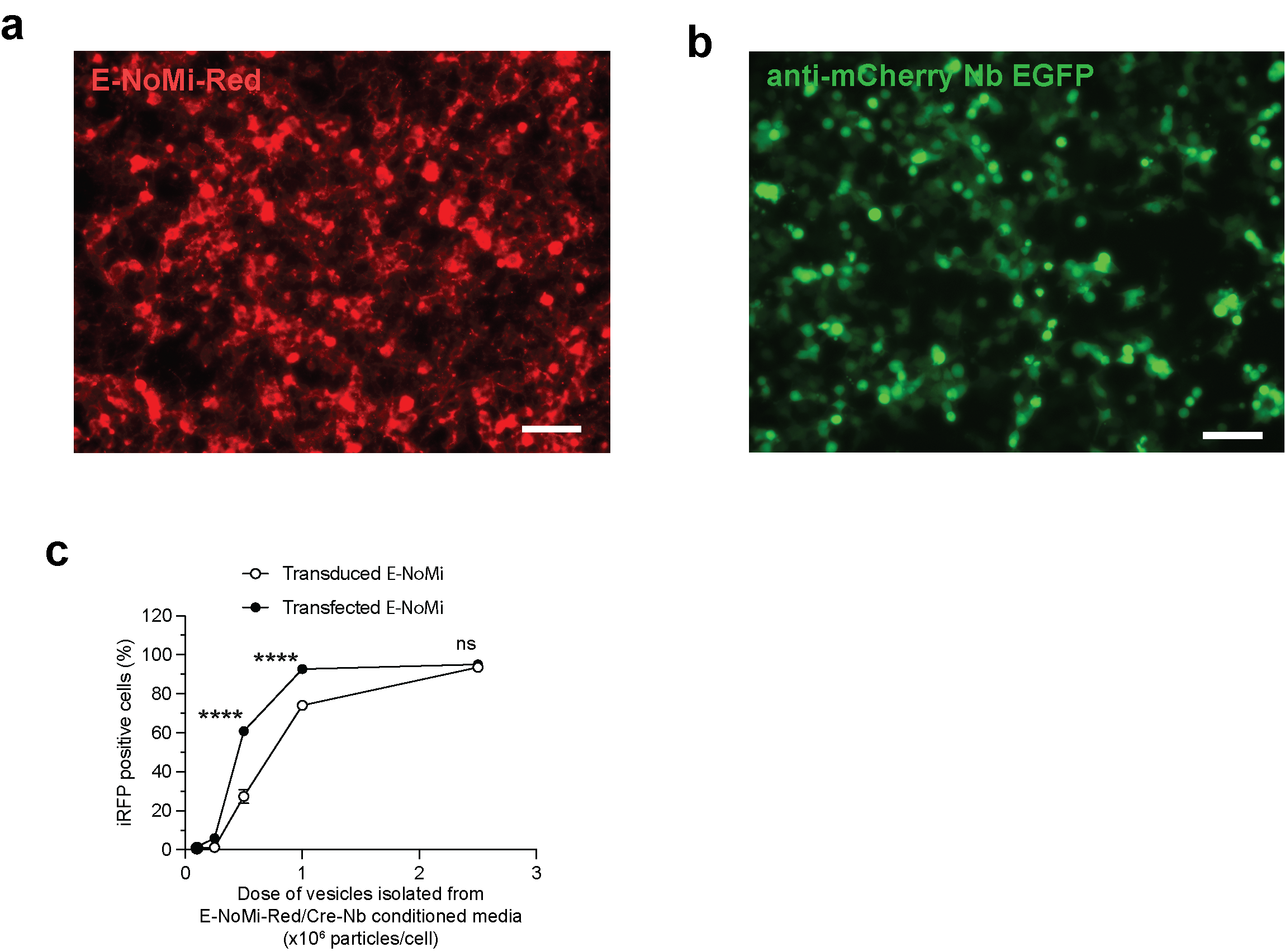


**Figure S7**

(A) Fluorescence microscopy of E-NoMi-Red (mCherry^POS^) expressing cells, 99% of cells expressed mCherry. The scale bar represents 50 µm.

(B) Florescence microscopy of anti-mCherry Nb EGFP (EGFP^POS^) expressing cells, 95% of cells expressed EGFP. The scale bar represents 50 µm.

(C) Flow cytometry assessment of EVV Cre delivery comparing transiently expressed E-NoMi-Red producer cells to stably expressing E-NoMi-Red producer cells showing increased delivery from transiently expressed producer cells to Cre-reporter cells.

Anti-mCherry nanobody (Nb) gBlock:

tTCGTGACCGCCGCCGGGATCACTCTCGGCATGGACGAGCTgTACAAGGAGAACCTCTACTTTCAGTCCGGCGCGCCTATGGCCCAGGTGCAGCTGGTGGAGAGCGGCGGCGGCCTGGTGCAGGCCGGCGGCAGCCTGAGACTGAGCTGCGCCACCAGCGGCTTCACCTTCAGCGACTACGCCATGGGCTGGTTCAGACAGGCCCCCGGCAAGGAGAGAGAGTTCGTGGCCGCCATCAGCTGGAGCGGCCACGTGACCGACTACGCCGACAGCGTGAAGGGCAGATTCACCATCAGCAGAGACAACGTGAAGAACACCGTGTACCTGCAGATGAACAGCCTGAAGCCCGAGGACACCGCCGTGTACAGCTGCGCCGCCGCCAAGAGCGGCACCTGGTGGTACCAGAGAAGCGAGAACGACTTCGGCAGCTGGGGCCAGGGCACCCAGGTGACCGTGAGCAAGGAGGCCATCTGATCGACAATCAACCTCTGGATTACAAAATTTGTGAAAGATT

Anti-ALFA tag Nb gBlock:

tTCGTGACCGCCGCCGGGATCACTCTCGGCATGGACGAGCTgTACAAGGAGAACCTCTACTTTCAGTCCGGCGCGCCTGTGCAGCTGCAGGAGAGCGGCGGCGGCCTGGTGCAGCCCGGCGGCAGCCTGAGACTGAGCTGCACCGCCAGCGGCGTGACCATCAGCGCCCTGAACGCCATGGCCATGGGCTGGTACAGACAGGCCCCCGGCGAGAGAAGAGTGATGGTGGCCGCCGTGAGCGAGAGAGGCAACGCCATGTACAGAGAGAGCGTGCAGGGCAGATTCACCGTGACCAGAGACTTCACCAACAAGATGGTGAGCCTGCAGATGGACAACCTGAAGCCCGAGGACACCGCCGTGTACTACTGCCACGTGCTGGAGGACAGAGTGGACAGCTTCCACGACTACTGGGGCCAGGGCACCCAGGTGACCGTGAGCAGCTCGACAATCAACCTCTGGATTACAAAATTTGTGAAAGATT

TEV-containing handle gBlock:

GTGGGTGTCGGGGCACAGCTTGTCCTGAGTCAGACCATATCTAGAATTCCTAGGGAGAACCTCTACTTTCAGTCCGACTACAAAGACCATGACGGTGATTATAAAGATCATGACATCGATTACAAGGATGACGATGACAAGGGTGGTTCTGGTGGTGGTTCTGGTCGATCCACCATGGTCTTCACACTCGAAGATTTCGTTGGGGACTGGCGACAGACAGCCGGCTACAACCTGGACCAAGTCCTTGAACAGGGAGGTGTGTCCAGTTTGTTTCAGAATCTCGGGGTGTCCGTAACTCCGATCCAAAGGATTGTCCTGAGCGGTGAAAATGGGCTGAAGATCGACATCCATGTCATCATCCCGTATGAAGGTCTGAGCGGCGACCAAATGGGCCAGATCGAAAAAATTTTTAAGGTGGTGTACCCTGTGGATGATCATCACTTTAAGGTGATCCTGCACTATGGCACACTGGTAATCGACGGGGTTACGCCGAACATGATCGACTATTTCGGACGGCCGTATGAAGGCATCGCCGTGTTCGACGGCAAAAAGATCACTGTAACAGGGACCCTGTGGAACGGCAACAAAATTATCGACGAGCGCCTGATCAACCCCGACGGCTCCCTGCTGTTCCGAGTAACCATCAACGGAGTGACCGGCTGGCGGCTGTGCGAACGCATTCTGGCGGAGAACCTCTACTTTCAGTCCGGCGCGCCTTCTAGAATCCAGGGGGCTACCCCTGGCTCTCTGTTGCCAGTGGTC

Table 1: RT-qPCR primers

|  | Forward | Reverse |
| --- | --- | --- |
| Cre-Reporter ON | ACCGTCAGATCGCCTGG | CAAGAGGTCAGGCTGCCT |
| Cre-Reporter OFF | ACCGTCAGATCGCCTGG | CCTTAATCAGCTCGCTCATGGT |
| Beta-Actin | GTGACGTTGACATCCGTAAAGA | GCCGGACTCATCGTACTCC |
| GAPDH | GTCTCCTCTGACTTCAACAGCG | ACCACCCTGTTGCTGTAGCCAA |

Table 2: Western blot antibodies

| Protein | Antibody | Vendor |
| --- | --- | --- |
| CD81 | CD81 (D3N2D) Rabbit mAb #56039 | Cell Signalling Technologies |
| Flotillin-1 | Flotillin-1 Antibody #3253 | Cell Signalling Technologies |
| Calnexin | Calnexin Antibody #2433 | Cell Signalling Technologies |
| FLAG Tag | Monoclonal ANTI-FLAG® M2 antibody produced in mouse | Millipore Sigma |
| Cre | Cre Recombinase (D7L7L) XP® Rabbit mAb #15036 | Cell Signalling Technologies |

Table 3: sgRNA sequence

| Gene | Spacer Sequence | Sequence with **PAM** |
| --- | --- | --- |
| *EMX1* | GCAACCACAAACCCACGAGGG | GCAACCACAAACCCACGAGGG**CAGAGT** |
